# Bayesian Optimized High‐Figure‐of‐Merit Broadband Directional Thermal Emitters

**DOI:** 10.1002/nap2.70003

**Published:** 2026-01-14

**Authors:** Erwei Gui, Guangji Lian, Shenghao Jin, Jiahao Zhou, Shuai Gong, Changying Zhao, Boxiang Wang

**Affiliations:** ^1^ Institute of Engineering Thermophysics, School of Mechanical Engineering Shanghai Jiao Tong University Shanghai China; ^2^ 2020 X‐Lab Shanghai Institute of Microsystem and Information Technology Chinese Academy of Sciences Shanghai China; ^3^ State Key Laboratory of Transducer Technology Shanghai Institute of Microsystem and Information Technology Chinese Academy of Sciences Shanghai China

**Keywords:** Bayesian optimization, directional thermal emission, epsilon‐near‐zero (ENZ), infrared deception

## Abstract

Broadband directional thermal emitters have attracted significant attention due to their potential applications in infrared camouflage and radiative cooling. However, existing broadband directional thermal emission (BDTE) multilayer structures rely heavily on the Berreman modes of epsilon‐near‐zero (ENZ) materials, usually requiring a substantial number of stacked ENZ thin films for broader spectral coverage. Moreover, the lack of optimized thicknesses fails to achieve the optimal figure of merit (FOM) of BDTE. Here, we have realized a high‐FOM BDTE structure with a reduced number of ENZ layers based on Bayesian optimization. By coupling epsilon‐near‐pole (ENP) resonance with the Brewster effect of the dielectric spacer, we extend the BDTE bandwidth by 2 μm (from 7.9–12 to 7.9–14 μm). The optimized structure shows unprecedented performance, achieving an average directional emissivity of 0.94 and an FOM of 8.087, which are also validated by experimental measurements. Notably, by integrating our emitter with low‐emissivity covers, we develop a series of patterned devices for infrared information encryption and deception applications, which exhibit angle‐dependent distinct, even contradictory, infrared information. This work not only provides theoretical guidance for the design and optimization of BDTE structures but also paves the way for their applications in infrared information technologies.

## Graphical abstract

By leveraging ENP resonance and Bayesian optimization, a high‐FOM broadband directional thermal emission covering the wavelength range of 8–14 μm is achieved by four epsilon‐near‐zero (ENZ) material layers. Moreover, a series of patterned devices are developed for infrared information encryption and deception applications, which exhibit distinct, even contradictory, infrared information under normal and oblique directions.

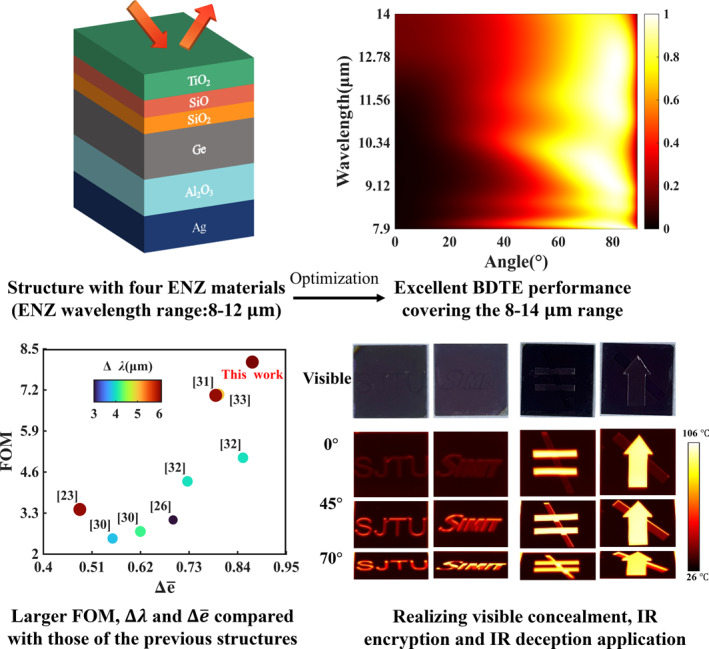

## 1 Introduction

Thermal radiation is ubiquitous in the universe as an important carrier of energy and information. Typically, the thermal radiation of common objects is broadband, unpolarized, and omnidirectional [1], which usually leads to information leakages and energy losses at undesired directions, rendering the high‐efficiency utilization of thermal radiation energy very challenging. Directing thermal emission to specific directions has been realized by a variety of different nanophotonic structures, such as photonic crystals [2, 3], gratings [4, 5, 6], and metasurfaces [7, 8]. However, the optical resonances that direct thermal emission in these structures strongly depend on the wavelength and the emission angle, usually resulting in narrowband directional thermal emission, with the direction of emission changing significantly with wavelength [4, 8, 9]. It is thus very challenging to realize broadband and directional manipulation of thermal radiation, which is of particular importance for emerging applications such as infrared camouflage [10, 11, 12], infrared encryption [13, 14], and radiative cooling [15, 16, 17, 18, 19, 20].

Recently, since thin films based on ENZ materials support leaky *p*‐polarized electromagnetic resonances, which exhibit strong absorption/emission at wavelengths where the permittivity approaches zero at certain incident angles, known as Berreman mode [21], multilayer ENZ films have been proposed to realize broadband directional thermal emission (BDTE) [22, 23, 24, 25, 26]. In these designs, a highly reflective metal substrate [27, 28] is typically used to enhance angle‐dependent absorption induced by the Berreman mode and effectively suppress omnidirectional absorption caused by transverse optical (TO) phonon resonance [29]. For instance, Xu et al. [30] designed two samples with different ENZ materials (Al_2_O_3_, SiO_2_, SiO; TiO_2_, MgO, Ta_2_O_5_) sequentially stacked on the metal substrate. These designs achieved BDTE in the wavelength ranges of 7.7–11.5 and 10.0–14.3 μm, respectively, with both exhibiting a maximum averaged directional emissivity of about 0.70. Mahato et al. [25] investigated the BDTE performance of four ENZ materials (SiO, SiN, Al_2_O_3_, Ta_2_O_5_) stacked on the metal substrate with different sequences and thicknesses and proposed a multilayer structure achieving BDTE in 8–13 μm with a maximum averaged directional emissivity of about 0.78. However, it is difficult for ENZ multilayer structures directly stacked on metal substrates to realize a spectrally averaged directional emissivity exceeding 0.9 [23, 24, 25, 26, 30]. More importantly, since each natural ENZ material supports only one unique Berreman mode wavelength, it is usually necessary to stack a variety of different ENZ materials (covering 8–14 μm typically requires at least five ENZ materials [31]) to achieve broader spectral coverage, leading to increased manufacturing complexity and high cost. Therefore, it is still challenging to realize high‐performance, broader‐bandwidth BDTE using a relatively small number of ENZ thin film layers. Moreover, extending the bandwidth of BDTE by increasing the number of ENZ layers in multilayer films on metal substrates tends to result in a large total thickness, which would diminish the field confinement effect [31] within ENZ layers and inevitably excite unwanted TO resonances, leading to a deterioration of BDTE performance (Figure S1a). Introducing a lossless dielectric material such as Ge [31, 32] or a‐Si [33] into the ENZ multilayer film structure, via the coupling between Fabry–Pérot (FP) modes and Berreman modes, can partially solve this issue. However, existing works typically lack optimized structural thicknesses, and thus fail to achieve the optimal figure of merit (FOM) of BDTE over the entire desired wavelength range.

In this work, to address current challenges in BDTE, we design a high‐FOM broadband directional thermal emitter covering the entire long wave‐infrared (LWIR, 8–14 μm) band with a reduced number of ENZ materials based on the Bayesian optimization (BO) strategy. We demonstrate that coupling the epsilon‐near‐pole (ENP) resonance of ENZ materials with the Brewster effect of the dielectric spacer can significantly extend the BDTE bandwidth by 2 μm (from 7.9–12 to 7.9–14 μm) without increasing the number of ENZ materials, thus relaxing the requirement for ENZ materials. By employing BO to identify the optimal thicknesses from $1.178\times 10^{10}$ candidates of thickness combinations with relatively low computational loads, we obtain a multilayer structure composed of TiO_2_/SiO/SiO_2_/Ge/Al_2_O_3_ on a metal substrate, exhibiting the largest FOM (∼8.087) to date, which achieves high spectral average emissivity contrast of about 14:1 between 79° (${\bar{e}}_{7.9-14\mu m}$∼0.94) and 0° (${\bar{e}}_{7.9-14\mu m}$∼0.067) in *p* polarization, implying excellent BDTE performance. We fabricate the designed broadband directional thermal emitters, whose experimental spectra agree well with theoretical calculations. Moreover, by integrating our broadband directional thermal emitter with low‐emissivity covers exhibiting similar structural colors, we develop a series of patterned devices for infrared information encryption and deception applications. These devices exhibit distinct, even contradictory, infrared information under normal and oblique directions when observed via an infrared camera. Notably, we also demonstrate that the designed structure can be deposited on flexible, large‐scale substrates of various types, with an overall thickness of only 1.69 μm and strong manufacturing robustness, significantly expanding the application scenarios. This work not only provides theoretical guidance for the design and optimization of BDTE structures but also paves the way for their applications in infrared information technologies.

## 2 Results and Discussions

### 2.1 Material Selection and Design Principle

According to the generalized Fresnel formula, the reflectance of an ultrathin ENZ layer stacked on a metal exhibits a pronounced minimum for specific ENZ film thicknesses [27]:

(1) hB=λBerreman2πcosθsin2θIm−1εmax−1,

where ε is the complex permittivity of the material, $\lambda_{\text{Berreman}}$ refers to the Berreman wavelength of ENZ materials, which corresponds to the maximum value of Im{−1/ε}, and θ is the emission angle. Since the excitation of the Berreman mode is highly dependent on the confinement of the longitudinal electric field component $E_{z}$, the directional emission angle of BDTE designs based on ENZ materials mainly falls within a large angle range in *p* polarization. However, the Berreman thickness of each ENZ material calculated at a given emission angle, when directly applied to the multilayer film structure for BDTE design, cannot deliver optimal performance across the entire designed wavelength range (especially in non‐ENZ wavelength ranges), thus requiring further optimization of the thickness of each material layer (Figure S1a,b).

**FIGURE 1:**
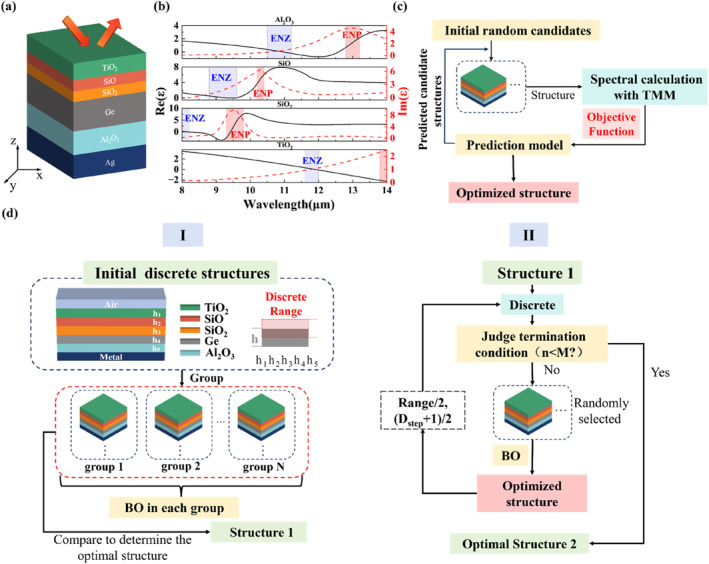
(a) Schematic of the designed BDTE structure. (b) Permittivities of different ENZ materials selected in this work [34, 35]. The black and red shaded areas represent ENZ and ENP wavelength ranges, respectively. (c) Schematic of the Bayesian optimization (BO) method combined with the transfer matrix method (TMM). (d) Flowchart for the optimization strategy of BDTE adopted in this work. Process I (left) obtains optimized Structure 1 by performing BO on each of the 2000 groups divided from the initial 1.178 × 10^10^ candidate structures, whereas Process II (right) achieves finer optimization of Structure 1 to obtain optimal Structure 2 by continuously reducing discrete step size and ranges.

The selected materials in this work and the designed stacking sequence of each material are shown in Figure 1a. A structure with four ENZ materials stacked with a dielectric spacer on a metal substrate, whose stacking sequence is TiO_2_/SiO/SiO_2_/Ge/Al_2_O_3_/Ag (from top to bottom), is proposed to realize high‐performance BDTE over the 8–14 μm broadband range. This structural stacking sequence is derived from analyzing the longitudinal electric field distributions of various candidate stacking sequences (Figure S16). The corresponding Berreman wavelengths of four selected ENZ materials (TiO_2_, SiO, SiO_2_, and Al_2_O_3_) are 11.74, 8.83, 8.03, and 10.68 μm, respectively (Figure 1b). Moreover, due to the ENP resonance, Al_2_O_3_ and TiO_2_ exhibit strong absorption in the 12–14 μm range, whereas SiO_2_ and SiO exhibit high absorption in the 9–10 μm range (red shaded areas in Figure 1b). It is evident that relying solely on the Berreman modes of the ENZ materials cannot achieve BDTE that covers the entire LWIR band. To address this limitation, we utilize the coupling between the ENP resonance of the ENZ materials and the Brewster effect of the Ge layer to further expand the wavelength range of BDTE. The Brewster effect of high‐refractive‐index dielectric materials exhibits near‐zero reflectivity at the Brewster angle $\theta_{B}$ over a broad wavelength range [36, 37]. This effect, like the Berreman mode, can only be excited under *p* polarization and is highly angle‐dependent. By setting the *p*‐polarized reflection coefficient to zero, the Brewster angle $\theta_{B}$ of a freestanding Ge layer can be calculated as [37]

(2) $$\begin{array}{c} \theta_{B}=\sin^{-1}\left(\frac{n_{1}n_{\text{Ge}}}{n_{0}\sqrt{n_{1}^{2}+n_{\text{Ge}}^{2}}}\right), \end{array}$$

where $n_{1}$, $n_{\text{Ge}}$, and $n_{2}$ are the refractive indices of the incident medium, the Ge layer, and the exit medium, respectively. The Brewster angle of the Ge layer in air is about 76° in the wavelength range of 8–14 μm.

For *p*‐polarized incident light, the combination of the Brewster effect and the FP mode in the Ge layer exhibits high transmittance near the Brewster angle $\theta_{B}$ (> 60°) while demonstrating high reflection at angles < 60° across the LWIR band (Section S3 and Figure S2a). When coupled with the omnidirectional and broadband absorption induced by ENP resonance in multilayer ENZ materials, this Ge layer suppresses absorption within small incident angles (< 60°) while leaving absorption near the Brewster angle unaffected. In our design, for large incident angles at *p* polarization, owing to the coupling of the Berreman modes with the FP mode in the Ge layer, the longitudinal electric field component $E_{z}$ is still strongly confined at the Berreman wavelengths of each ENZ material, covering the 8–12 μm wavelength range. This large field localization induces strong absorption, even if the ENZ materials exhibit relatively low loss at these wavelengths. Meanwhile, the FP cavity (Ge layer) facilitates the suppression of undesired absorption caused by TO resonance at small angles by changing the spatial field distribution of the structure [31, 32]. Within the wavelengths of 12–14 μm, the ENP resonances of TiO_2_ and Al_2_O_3_ layers show omnidirectional absorption. In this circumstance, *p*‐polarized incident light at large angles (near the Brewster angle of the Ge layer) will be absorbed in the TiO_2_ and Al_2_O_3_ layers. In contrast, at small incident angles, the absorption caused by ENP resonance is inhibited due to the high reflectance of the Ge layer. By judiciously designing the layer thickness to make the directional emission angle of the Berreman modes coincide with the Brewster angle of the Ge layer, our structure can realize high‐performance BDTE covering the LWIR band under *p* polarization. In addition, since neither the Brewster effect nor the Berreman modes can be supported under *s* polarization, the structure exhibits omnidirectional high reflectance throughout the entire LWIR band. Notably, the ENP resonance wavelengths of ENZ materials should be contiguous with the ENZ wavelength range to ensure a continuous BDTE band. Besides the materials selected in this work, other ENZ materials such as Ta_2_O_5_, AlN, SiN, and MgO (Figure S15) and lossless dielectric materials such as Si and ZnS exhibit great potential for expanding the BDTE wavelength range.

### 2.2 Bayesian Optimization

Traditional optimization methods such as genetic algorithms [38] and particle swarm optimization [39] for the design of multilayer structures typically require the calculation of the objective function for an enormous number of candidate structures (exploitation), reducing optimization efficiency. BO avoids this “exploitation‐only” drawback through efficient machine learning and prediction, thus identifying the optimal structures among the enormous candidate structures with low computational effort and high efficiency [40, 41]. Existing works, such as the optimization for narrowband selective thermophotovoltaic emitters and ultranarrow‐band selective thermal emission [42, 43, 44], have demonstrated that BO can achieve the optimal balance between exploration and exploitation. As the number of candidate structures grows exponentially with increasing film layer numbers in multilayer structures, BO displays greater applicability and efficiency. In our design, the thickness of each layer is optimized using PHYSBO [45, 46], which is based on the COMmon Bayesian Optimization (COMBO) library.

Figure 1c shows the schematic of a single Bayesian optimization process combined with the transform matrix method (TMM, Section S1). *N*_r_ initial random candidates, which are selected from a large number (*N*_t_) of initially discretized candidates, together with their corresponding values of the objective function (OF) calculated by TMM, serve as training data for the Gaussian prediction model. The prediction process relies on the probabilistic surrogate model and the acquisition function [40]. Through this process, a new variable candidate whose OF value exceeds the maximum OF value within the database is proposed and integrated into the database to update the Gaussian prediction model. Finally, the prediction process is performed *N*_p_ times, and the structure corresponding to the maximum OF value is selected from the final database as the optimized structure. The OF for BDTE performance is defined as

(3) $$\text{OF}=f_{1}\sum_{i=1}^{M_{1}}\bar{e}\left(\alpha_{i}\right)-f_{2}\sum_{j=1}^{M_{2}}\bar{e}\left(\beta_{j}\right),$$

(4) $$\begin{array}{c} \bar{e}\left(\theta \right)=\frac{\int_{\lambda_{0}}^{\lambda_{1}}e\left(\lambda ,\theta \right)d\lambda }{\lambda_{1}-\lambda_{0}}, \end{array}$$

where e(λ,θ) refers to the spectral directional emissivity in *p* polarization calculated by TMM. $\lambda_{0}$ and $\lambda_{1}$ are the maximum and minimum wavelengths for optimization, and $\lambda_{0}=8\;\mu m$, $\lambda_{1}=14\;\mu m$. $\bar{e}$ is the averaged directional emissivity, θ is the emission angle and θ∈[0,π/2]. $\alpha_{i}$ and $\beta_{j}$ are the large and small emission angles, which represent the desired and undesired emission directions, respectively. To reduce the optimization load, we uniformly discretize the desired and undesired emission angle ranges, where *M*_1_ and *M*_2_ correspond, respectively, to the number of discrete angular values for these two ranges. The desired angular range falls within 60°–90° due to the Brewster effect of the Ge layer. *f*_1_ and *f*_2_ represent the weights for emissivity in the desired and undesired emission angles, respectively. When *f*_1_ > *f*_2_, the optimization result will favor a larger average emissivity at large angles. The BO process yields an optimal structure that seeks to meet the desired performance to the greatest extent by maximizing the OF. In this optimization process, the angular ranges are $\alpha_{i}\in \left[70{}^{\circ},85{}^{\circ}\right]$, $\beta_{j}\in \left[5{}^{\circ},30{}^{\circ}\right]$, the weight values are *f*_1_ = 0.6 and *f*_2_ = 0.4, and the discrete angular numbers are *M*_1_ = *M*_2_ = 25, all of which are chosen to achieve excellent BDTE performance.

Figure 1d presents the optimization strategy adopted in this work. The optimization process, based on the initial thickness, generates numerous discrete thickness combinations within discrete ranges as initial candidate structures, and the precision of the process is determined by the discrete step size. The discrete thicknesses of each material layer are represented by variables $h_{1}$–$h_{5}$, as the discrete thickness sets of each layer from top to bottom. To ensure sufficiently precise outcomes and avoid overlooking better thickness combinations, the optimization process is divided into two stages: Process I and Process II.

**FIGURE 2:**
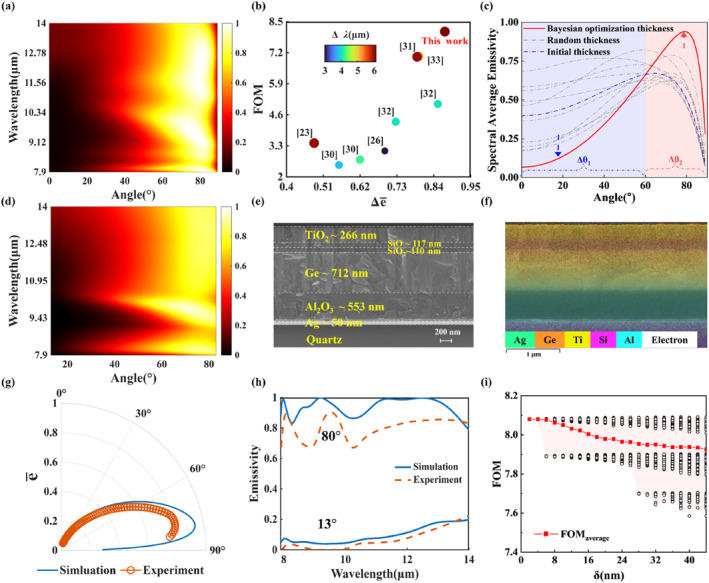
(a) Simulated emissivity spectrum of the designed BDTE emitter under *p* polarization with varying incident angles. (b) Comparison of FOMs for existing broadband directional thermal emitters. (c) Simulated spectral average emissivity of the optimal structure (red solid line), different structures with random thicknesses (gray dashed line), and the structure with initial thicknesses (blue dashed line) before optimization under *p* polarization varying with emission angles. (d) Measured absorptivity spectrum of the fabricated BDTE sample under *p* polarization with varying incident angles. (e) SEM image of the fabricated BDTE sample. (f) EDS image of the fabricated BDTE sample. (g) The simulated and experimental average emissivity $\bar{e}$ of the designed BDTE structure varying with emission angles in *p* polarization. (h) Comparison of simulated (solid line) and experimental (dashed line) emissivity spectra for emission angles of 13° and 80° under *p* polarization. (i) FOM of the designed structure varying with thickness deviation range δ of each layer. The black open circles in the figure represent the FOMs from 800 random thickness calculations within the corresponding thickness deviation range.

Process I optimizes the layer thicknesses within the large ranges using a 6‐nm discrete step size, ensuring ample optimization ranges and a sufficient number of candidate structures. The discrete thickness ranges of each material are 600, 500, 500, 1000, and 600 nm, respectively, and the number of initial possible candidates is $100\times 84\times 84\times 167\times 100\approx 1.178\times 10^{10}$. To demonstrate the universality of the optimization method, we arbitrarily choose the initial thicknesses of layers as 500, 300, 300, 1000, and 600 nm (from top to bottom). Additionally, as verified through multiple optimization tests, other random initial thicknesses (gray lines in Figure 2c) can also converge to the final optimal thicknesses. Since the number of initial structures is so large that the BO process requires an excessive amount of memory, the candidate structures are sequentially divided into 2000 groups, with about $5.89\times 10^{6}$ initial possible structures in each group, to reduce computational loads, and a separate BO process is performed for each group. In this process, the numbers of initial random and predicted candidate structures are set to 165 and 10, respectively, with the verification of optimization stability (Figure S3a,b). Following the completion of Process I, the global best optimized Structure 1, with a specific thickness distribution of 242, 104, 92, 692, and 542 nm for each layer (from top to bottom), is identified from these 2000 locally optimized structures (Figures S4 and S6) and adopted as the initial structure for Process II, which operates with a higher optimization precision. The total computational time of Process I is about 8 days with 128 GB of computational memory and a 13th Gen Intel Core i9‐13900K processor (32 cores).

To obtain a more refined optimization result while avoiding the omission of potential effective structures, Process II employs a strategy of progressively reducing the discrete ranges and the step size to further refine the optimized Structure 1 from Process I. The initial discrete ranges for each layer are set to 200, 100, 100, 300, and 200 nm, respectively, with an initial discrete step size of 6 nm. Additionally, the numbers of initial random candidate structures and predicted candidate structures are set to 200 and 30, respectively. As the optimization proceeds, the optimized result from each BO iteration serves as the initial structure for the subsequent iteration, which is performed with smaller discrete ranges and a smaller discrete step size. Notably, the discrete step size remains unchanged once it reaches 1 nm, whereas the discrete thickness ranges continue to decrease, leading to a progressive decrease in the number of initial discrete candidates (Figure S5a). Separately, Process II is terminated when the number of initial discrete structures falls below 1000. Therefore, as the number of BO iterations increases and the number of discrete candidate structures accordingly decreases, the optimization process is completed once this threshold is met, yielding the final optimal Structure 2. Process II has completed seven iterations in this optimization, and the maximum OF value stabilizes after the fourth iteration (Figure S5b), which demonstrates that the final optimal result has been achieved.

The simulated emissivity spectrum of the multilayer structure with optimal thicknesses (251, 119, 93, 683, and 547 nm, from top to bottom) under *p* polarization is given in Figure 2a, showing excellent BDTE performance. To quantitatively evaluate the BDTE performance, we propose a figure of merit (FOM) as

(5) $$\begin{array}{c} F\text{OM}=\Delta \lambda \cdot \left(\Delta \bar{e}/\Delta \theta \right), \end{array}$$

where $\bar{e}$ refers to the average directional emissivity calculated by Equation (4). $\Delta \bar{e}={\bar{e}}_{\max}-{\bar{e}}_{\min}$, which is the emissivity difference representing the gap between the maximum and minimum average directional emissivity. Δλ represents the directional wavelength range, which is defined as the wavelength range where $e\left({\lambda_{i},\theta }_{i}\right)\geq 0.8$ for any $\lambda_{i}$ within. Note that many structures in previous works for comparison cannot meet this condition even across the entire reported BDTE wavelength range [23, 26, 30, 31, 32, 33]. For these structures, Δλ is then defined as their claimed BDTE range, which would then overestimate their FOMs. Δθ is the angle range of directional emission and is defined as the angular range of $\theta_{i}$ that satisfies $\bar{e}\left(\theta_{i}\right)>{\bar{e}}_{\max}/2$. Better BDTE performance is achieved for a smaller Δθ, a broader Δλ, and a greater $\Delta \bar{e}$. Notably, when using this FOM as the optimization target, the optimization requires full angular range (0°–90°) emissivity data to calculate the FOM of a candidate structure, which increases the complexity of identifying the desired optimal structure and imposes higher requirements on computational memory and time. Moreover, the angle range of directional emission defined by the FOM varies with the candidate structures, which results in a lack of explicitness and clarity in the optimization objective, reducing the optimization efficiency and performance. Additionally, the FOM fails to balance high emission at large angular ranges and low emission at small angular ranges by tuning weight parameters. Therefore, this FOM is not suitable as an objective function for the BO process.

As shown in Figure 2b, the theoretical emissivity difference $\Delta \bar{e}$ and FOM of the optimized structure are 0.87 and 8.087, respectively, representing the highest performance among all previously reported multilayer BDTE structures [23, 26, 30, 31, 32, 33]. Our designed structure exhibits a spectral directional emissivity $e\left({\lambda_{i},\theta }_{i}\right)\geq 0.81$ over the wavelength range of 7.9–14 μm (which covers the LWIR band), and has an angle range of directional emission Δθ of 37.7°. Additionally, our structure realizes ${\bar{e}}_{7.9-14\mu m}∼0.069$ (at 0°) and ${\bar{e}}_{7.9-14\mu m}∼0.94$ (at 79°), which correspond to an average directional emissivity ratio of 1:14, demonstrating the superior BDTE performance. To further illustrate the efficacy of the optimization strategy, we compare the results of average emissivity for 10 groups of random thicknesses (all within the optimization discrete range), initial optimization thicknesses, and optimal thicknesses (Figure 2c). The average emissivity of the structure exhibits, after optimization, a significant reduction in the undesired emission angular range $\Delta \theta_{1}$ (0°–60°) while showing an increase in the desired emission angular range $\Delta \theta_{2}$ (60°–90°), indicating that the optimization process significantly and effectively enhances the structure's BDTE performance.

### 2.3 Experimental Characterization and Analysis of Mechanisms

The designed multilayer structure is fabricated by magnetron sputtering (DISC‐SP‐3200) on a quartz substrate with a size of 50 × 50 × 1mm^3^. According to Kirchhoff's law, the spectral angular emissivity e(λ,θ) equals its spectral angular absorptivity α(λ,θ), expressed as e(λ,θ)=α(λ,θ). In order to validate the performance of the fabricated emitter, the *p*‐polarized and *s*‐polarized angle‐resolved absorptivity spectra (Figure 2d and Figure S13) of the fabricated BDTE sample with an angular range of 13°–83° are measured by a Fourier transform infrared spectrometer (Vertex 70v, Bruker). The fabricated sample exhibits a significant difference in absorptivity/emissivity between small and large incident angles under *p* polarization, realizing excellent BDTE performance across the 7.9–14 μm range. The scanning electron microscope‐energy dispersive spectrometry (SEM‐EDS) images are presented in Figure 2e,f and Figure S7. The elemental position and distribution of each layer correspond well to the designed structure, verifying the high precision of the fabrication process. Moreover, the thicknesses of TiO_2_, SiO, SiO_2_, Ge, Al_2_O_3_, and Ag layers are 266, 117, 110, 712, 553, and 50 nm, respectively, which are measured by ellipsometry (Semilab SE‐2000) and match the optimally designed thicknesses closely. We further extract the emissivity spectra at two specific emission angles of 80° and 13°, which show good agreement with theoretical calculations (Figure 2h). The experimental emissivity at the angle of 13° keeps rather low (< 0.2) across the 7.9–14 μm range, demonstrating that the absorption/emission at small angles is successfully suppressed by the Ge layer. At the angle of 80°, due to thickness deviations in experimental fabrication and discrepancies in the material refractive indices, the experimental emissivity is slightly lower than the theoretically calculated emissivity. Nevertheless, the structure still exhibits strong emission in the 12–14 μm wavelength range (the non‐ENZ wavelength range) as well as the 8–12 μm range covered by the Berreman wavelengths of each ENZ material (namely, 11.74, 8.83, 8.03, and 10.68 μm, from top to bottom), which confirms that the BDTE wavelength range is significantly broadened by coupling the ENP resonance with the Brewster effect of the Ge layer. Due to experimental limitations, the test angle range (13°–83°) of spectral measurement cannot cover the entire polar emission angle range (0°–90°). However, the FOM of the fabricated emitter is still estimated and calculated to demonstrate its performance. The simulated and experimental average directional emissivity in the 7.9–14 μm range varying with the emission angle is shown in Figure 2g. Within the tested angular range, the experimental sample exhibits a $\Delta \bar{e}$ (emissivity difference) of 0.756 with ${\bar{e}}_{\max}∼$0.806 (at 77°) and ${\bar{e}}_{\min}∼$0.05 (at 13°) under *p* polarization, which is slightly lower than the theoretical result ($\Delta {\bar{e}}_{\text{theory}}∼$0.87). Moreover, the angle range of directional emission Δθ of this sample is 34° (smaller than $\Delta \theta_{\text{theory}}$ ∼ 37.7°). Though the fabricated emitter cannot meet the condition defined for the theoretical directional wavelength range, any $\lambda_{i}$ within the 7.9–14 μm range satisfies $e\left({\lambda_{i},\theta }_{i}\right)\geq 0.68$ and the average emissivity for maximum spectral emissivity in this range remains as high as approximately 0.822 (Figure S14). As such, the employed directional wavelength range Δλ of the FOM is still 6.1 μm. Consequently, the estimated FOM is 7.769 which is slightly lower than the theoretical result (FOM ∼8.087). Nevertheless, it still exhibits excellent performance among all previous structures in Figure 2b, demonstrating that our fabricated sample can achieve excellent BDTE performance. Additionally, the nonpolarized average emissivity contrast of our sample is ∼7.4:1 (Figure S17), which also exhibits great potential for practical applications.

To investigate the effects of thickness deviations on BDTE performance in the experimental processing of multilayer films, we randomly select the thickness of each layer within a deviation range of δ from its optimal thickness and calculate the FOMs of structures with these random thickness combinations. This process is performed by repeating random calculations for 800 times, and the corresponding FOM varying with δ is shown in Figure 2i. Within the thickness deviation range of 44 nm (namely, ± 22 nm from the optimal thickness), the reduction from the optimal FOM remains < 0.5, the structure maintains a high FOM (> 7.6), and the average FOM of the 800 random calculations is above 7.9 (higher than that in previous works), which demonstrates that the designed structure exhibits excellent robustness against thickness variations.

In order to further elucidate the mechanism of high‐FOM BDTE performance of our structure, we calculate the electric field and the bulk absorption at 80° incidence in *p* polarization for the optimized structure. The electric field is calculated by the Lumerical's STACK optical solver, and the quantitative absorption is calculated as

(6) $$\begin{array}{c} A\left(\lambda ,z\right)=\frac{4\pi \kappa \left(\lambda \right)n\left(\lambda \right)}{\lambda }|\frac{E\left(\lambda ,z\right)}{E_{0}\left(\lambda \right)}{|}^{2}, \end{array}$$

where *z* is the position in the thickness direction, and *n* and κ are the refractive index and extinction coefficient, respectively. The partial absorption of each layer is calculated by integrating A(λ,z) over the entire thickness of the corresponding layer.

**FIGURE 3:**
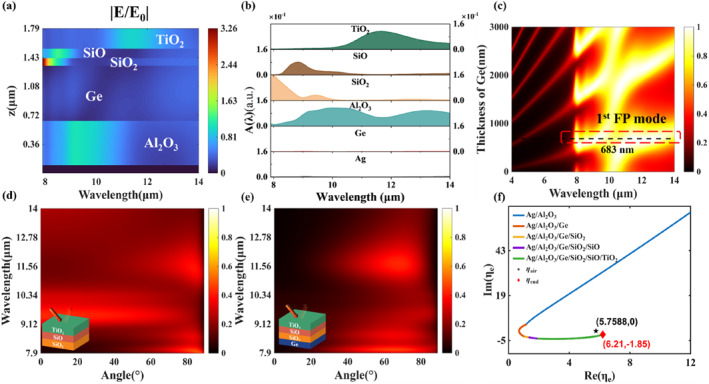
(a) Electric field distributions across the *z* direction at different wavelengths for *p* polarization at an incident angle of 80°. (b) Partial absorption of each layer at different wavelengths for *p* polarization at an incident angle of 80°. (c) Emissivity spectrum of the optimal structure varying with thickness of the Ge layer and wavelength at an emission angle of 80°. (d) Angle‐resolved emissivity spectrum of the upper three ENZ material layers with optimized thickness under *p* polarization. (e) Angle‐resolved emissivity spectrum of the upper three ENZ material layers after stacking with Ge at the bottom with optimized thickness under *p* polarization. (f) The admittance trajectory of the multilayer structure when stacked sequentially under *p* polarization and 80° incidence at the wavelength of 13 μm.

As shown in Figure 3a, the electric field in each ENZ material exhibits significant enhancement at its Berreman wavelength for 80° incidence, compared with that in the case of small incident angles (Figure S8a), indicating that the Berreman mode is supported in each layer. Moreover, due to the low‐reflection induced by the Brewster effect at large incident angles, the electric field is slightly enhanced in the TiO_2_ and Al_2_O_3_ layers across the non‐ENZ wavelength range. We further investigate the partial absorption of each layer at 80° incidence. As shown in Figure 3b, each ENZ material exhibits strong absorption at its Berreman wavelength and collectively gives rise to the high absorption at large incident angles in the 8–12 μm range. In addition, the small wavelength‐dependent slope of Al_2_O_3_'s permittivity real part, which still approaches zero near its Berreman wavelength [47], further augments absorption in the 8–12 μm range (Figure 1b). Within the 12–14 μm range, the relatively thick TiO_2_ and Al_2_O_3_ layers exhibit sufficient absorption due to their large imaginary parts of the permittivity (ENP resonance), achieving high absorption at large incident angles (Figure S8b).

In order to examine the role of the Ge layer in the multilayer structure, the *p*‐polarized emissivity spectrum varying with the thickness of the Ge layer at an emission angle of 80° is calculated (Figure 3c). The designed structure exhibits strong emission across the entire LWIR band when the optimized thickness of the Ge layer (683 nm) corresponds to the first‐order FP mode (Figure 3c), which demonstrates that the FP mode in the Ge layer is supported across the entire designed wavelength range. The coupling of the Berreman modes with the FP mode within the 8–12 μm range results in strong large‐angle emission. Within the 12–14 μm range, the collective coupling of the ENP resonance with both the Brewster effect and the FP mode achieves strong large‐angle emission. To further clarify the directional emission related to ENP resonance, the *p*‐polarized absorption spectra of the top three‐layer ENZ structure (TiO_2_/SiO/SiO_2_) before and after stacking a Ge layer at the bottom are calculated (Figure 3d,e). The top three‐layer ENZ structure with optimized thicknesses exhibits omnidirectional absorption and transmission, where the absorption is caused by ENP resonance. However, for the *p*‐polarized incidence at small angles, the four‐layer structure, with a 683‐nm Ge layer stacked at the bottom, exhibits suppressed absorption and high reflection across the LWIR band (Figure S9a). In contrast, the transmission and absorption for incident light near the Brewster angle are barely affected, revealing the strong coupling between the ENP resonance and the Brewster effect. Moreover, owing to the large imaginary part of its permittivity (near ENP resonance), the relatively thick Al_2_O_3_ layer with a metal substrate still exhibits substantial absorption in the 12–14 μm range (Figure S9b). Consequently, incident light near the Brewster angle, which transmits through the Ge layer, will be further absorbed in the bottom Al_2_O_3_ layer, achieving BDTE in the 12–14 μm range.

The effective admittance of the multilayer film (Section S2) can demonstrate the influence of each layer on the spectrum. The reflectivity of the multilayer structure can be calculated as [48]

(7) $$\begin{array}{c} R={\left(\frac{\eta_{0}-\eta_{e}}{\eta_{0}+\eta_{e}}\right)}^{2}, \end{array}$$

where $\eta_{e}$ and $\eta_{0}$ are the effective admittance of the multilayer structure and the admittance of the incident medium, respectively. The smaller the absolute difference between the effective admittance ($\eta_{e}$) and the admittance of the incident medium ($\eta_{0}$), the lower the reflectance of the multilayer film structure, corresponding to higher absorptivity/emissivity for the structure (if there is no transmission).

The admittance trajectory of the multilayer structure, as its layers are stacked sequentially, at 13 μm at an 80° emission angle in *p* polarization is shown in Figure 3f. A sufficiently thick Al_2_O_3_ layer enables the coordinate of the effective admittance ($\eta_{e}$) of the structure to continuously approach the real axis. When all film layers are stacked, the final effective admittance $\eta_{e}$ (6.21, −1.85) closely approaches the admittance of air $\eta_{a\text{ir}}$ (5.7588, 0) at 13 μm, realizing low reflectance and strong absorption (similar in other wavelengths of 9 and 11 μm, Figure S10). Moreover, the effective admittance of the multilayer structure is significantly closer to the admittance of air at an emission angle of 80° than at other incident angles (Figure S11), which further demonstrates that the directional thermal emission of the designed structure is concentrated near 80°.

### 2.4 Infrared Encryption and Deception Applications

**FIGURE 4:**
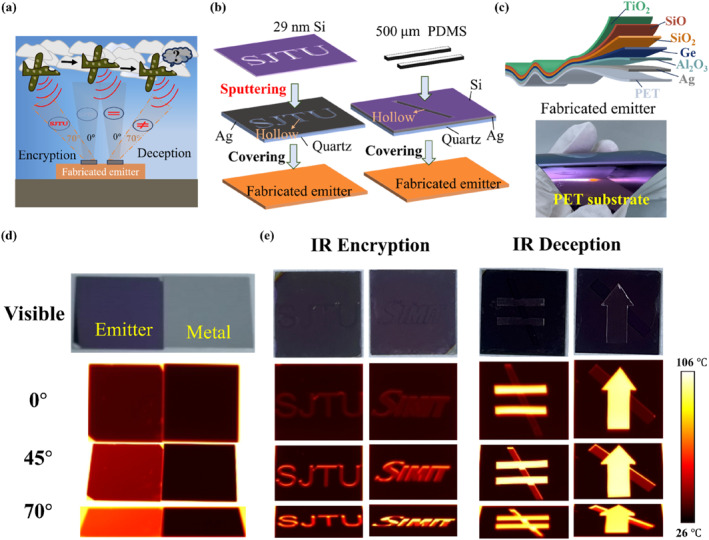
(a) Schematic of infrared encryption and infrared deception application scenarios. (b) Schematic of the designed covers for the infrared encryption and deception. (c) Schematic of the designed multilayer film stacked on a flexible PET substrate and the visible photograph of the fabricated flexible emitter (with a size of 50 × 50 mm^2^). (d) The visible and infrared images at different emission angles (0°, 45°, and 70°) of the fabricated emitter and the reference Ag sample. (e) The visible and infrared images of our patterned devices for encryption and deception at different emission angles (0°, 45°, and 70°).

Our BDTE structures have great potential in information security applications. As shown in Figure 4a, for an aircraft equipped with infrared (IR) detectors, an object integrated with broadband direction thermal emitters can achieve angle‐dependent information encryption and detector deception. For instance, no information and the pattern “=” can be detected at a normal observation angle, whereas the aircraft performs large‐angle detection, the information “SJTU” (abbreviated for “Shanghai Jiao Tong University”) and the illusory information featuring the pattern “≠” are detected, realizing the concealment and deception of IR information at different observation angles. As a proof‐of‐concept, we develop a series of patterned devices for infrared information encryption and deception applications, as shown in Figure 4b. A commercial quartz film with a size of 50 × 50 × 0.8mm^3^, featuring hollowed‐out “SJTU” pattern and “\” patterns, serves as the substrate for the cover. Then, an approximately 60‐nm thick silver (Ag) layer is deposited by magnetron sputtering to ensure high reflectance in the LWIR band. Subsequently, a 29‐nm thick silicon (Si) layer, which is optically lossless in the LWIR band, is further deposited on top of the Ag layer. This Si layer tunes the visible color of the cover to match that of the BDTE structures closely (Section S4 and Figure S12), achieving visible camouflage without reducing the reflectivity of the cover in the LWIR band. Moreover, the cover with hollowed‐out “\” patterns requires a thick polydimethylsiloxane (PDMS) layer laminated on its top surface, enabling the IR information display of the “=” pattern in the normal direction. Finally, these designed covers are covered on the BDTE samples to achieve IR encryption and IR deception. Similarly, we have designed and fabricated another cover featuring “SIMIT” (abbreviated for Shanghai Institute of Microsystem and Information Technology) and arrow patterns.

The schematic and visible photographs of our fabricated BDTE samples are shown in Figure 4c,d. The optimal structure is successfully deposited on a flexible polyethylene terephthalate (PET) substrate and a quartz substrate, each with a size of 50 × 50 mm^2^, verifying that our designed BDTE structure can be deposited on various types of flexible, large‐scale substrates with low manufacturing complexity. Since the sputtering materials employed in our emitters are common and readily available, further scaling‐up can be easily achieved through roll‐to‐roll magnetron sputtering [49, 50, 51], which facilitates practical applications. In addition, the high flexibility of the sample can further broaden its application scenarios in information communication. Our BDTE sample and the reference Ag sample are placed together on the hot stage with a heating temperature of 100°C to characterize the IR performance. The visible photograph and IR images at different emission angles (0°, 45°, and 70°) are shown in Figure 4d. The IR images are captured at different angles with an infrared camera, whose working wavelength range is 7.5–14 μm. At an emission angle of 0°, the BDTE sample exhibits low apparent temperature, which is similar to that of the metal sample. However, with the emission angle increasing, the apparent temperature of the sample increases and that of the metal still remains at room temperature, demonstrating that the fabricated emitter can effectively manipulate thermal radiation toward the desired large emission angles and also suppress emission in the undesired normal direction over the broadband wavelength range. At the emission angle of 70°, the apparent temperature of the BDTE sample is about 62°C, which is significantly higher than that of the metal (28°C).

The four covers featuring different hollowed‐out patterns are covered on the BDTE sample and positioned on a hot stage set to 100°C, constructing the IR encryption and deception devices (Figure 4e). For visible display performance, since all four covers exhibit good color matching with the BDTE sample, they achieve effective concealment of the visible information. Moreover, owing to the PDMS with a thickness of 500μm, our devices designed for IR deception exhibit patterns of equal sign “=” and arrow (indicating permission to pass), respectively, in visible images. Regarding IR encryption performance, it is hard to identify the information at a 0° emission angle. With the increasing emission angle, the patterns of “SJTU” and “SIMIT” grow progressively distinct, and these two pieces of information are easily observable at a 70° emission angle, achieving the angle‐dependent information concealment. For IR deception, due to the high emissivity of the PDMS, infrared images exhibit an “=” pattern and an arrow, respectively, at the 0° emission angle. Conversely, these two devices display an illusory inequality sign “≠” and a slash‐through arrow (indicating no passage) at a large emission angle, realizing angle‐dependent detector deception. Overall, our fabricated BDTE structures successfully enable IR deception and encryption while realizing effective concealment in visible images, demonstrating great potential for information communication technologies. In addition, since the Si layer is lossless in the LWIR band, directly stacking a Si layer on top of our emitters exhibits a certain coloration capability while retaining excellent BDTE performance (Figure S18), which can be further utilized in visible display applications.

## 3 Conclusion

In summary, we have realized a high‐FOM BDTE structure with a reduced number of ENZ layers based on Bayesian optimization. The experimental and theoretical results demonstrate that this design can broaden the BDTE bandwidth by 2 μm (from 7.9–12 to 7.9–14 μm) without increasing the number of ENZ materials due to the coupling of ENP resonance and the Brewster effect. In particular, our Bayesian‐optimized structure demonstrates the highest FOM (∼8.087) among existing BDTE multilayer structures and exhibits ${\bar{e}}_{7.9–14\mu m}∼0.069$ (at 0°) and ${\bar{e}}_{7.9–14\mu m}∼\;0.94$ (at 79°) in *p* polarization, corresponding to a great average directional emissivity ratio of 1:14. Moreover, we fabricate this BDTE structure and its measured results, with an emissivity difference $\Delta \bar{e}$ of 0.756, are in good agreement with theoretical calculations, demonstrating its experimental feasibility. We further investigate the impact of fabrication thickness deviation on device performance, and the results show that within a thickness deviation range of 44 nm, the designed structure can maintain highly robust performance. Notably, by integrating our emitter with low‐emissivity covers, we develop a series of patterned devices for infrared information encryption and deception applications, which exhibit angle‐dependent distinct, even contradictory, infrared information. This work not only provides theoretical guidance for the design and optimization of BDTE structures but also paves the way for their applications in infrared information technologies.

## 4 Experimental Section/Methods

### 4.1 Sample Fabrication

The BDTE multilayer samples and patterned covers are all fabricated using magnetron sputtering (DISC‐SP‐3200). The designed emitter is deposited on a quartz substrate with a size of 50 × 50 × 1 mm^3^. Meanwhile, the four covers employ quartz plates featuring designed hollowed‐out patterns as their substrates, and the sizes of the covers for IR encryption and IR deception are 50 × 50 × 0.8 mm^3^ and 60 × 60 × 0.8 mm^3^, respectively. Moreover, the 500‐μm thick polydimethylsiloxane (PDMS) layer is directly attached to the top of the covers for IR deception. For the fabrication process, argon (Ar) is employed as the working gas. The BDTE structure (TiO_2_/SiO/SiO_2_/Ge/Al_2_O_3_/Ag/quartz) and the cover (Si/Ag/quartz) are both stacked in accordance with the designed structural sequence and specified thicknesses. The processing parameters for each material are as follows:

The ENZ materials selected in our design are deposited via radio frequency (RF) magnetron sputtering. Silicon dioxide (SiO_2_) and silicon monoxide (SiO) operate at sputtering powers of 180 W (deposition rate ≈0.0585 nm s^−1^) and 150 W (deposition rate ≈0.1332 nm s^−1^), respectively, with an Ar flow rate of 40 sccm and an operating pressure of 0.3 Pa. Titanium dioxide (TiO_2_) operates at a sputtering power of 150 W (deposition rate ≈0.0137 nm s^−1^), with an Ar flow rate of 40 sccm, an O_2_ flow rate of 0.3 sccm, and an operating pressure of 0.3 Pa. Aluminum oxide (Al_2_O_3_) operates at a sputtering power of 200 W (deposition rate ≈0.0372 nm s^−1^), with an Ar flow rate of 30 sccm and an operating pressure of 0.27 Pa. Silver (Ag) and silicon (Si) are deposited via direct current (DC) sputtering, with an Ar flow rate of 40 sccm. Their operating pressures are 0.6 and 0.3 Pa, and sputtering powers are 100 W (deposition rate ≈0.6 nm s^−1^) and 150 W (deposition rate ≈0.148 nm s^−1^), respectively.

### 4.2 Emittance/Absorptance Spectrum Measurements

According to Kirchhoff's law, the spectral angular emissivity e(λ,θ) equals its spectral angular absorptivity α(λ,θ), expressed as e(λ,θ)=α(λ,θ). The angle‐resolved reflectivity of the fabricated sample is measured using a Fourier transform infrared spectrometer (FTIR; Vertex 70V, Bruker) with a ZnSe polarizer (WP25H‐Z, Thorlabs) and a reflection accessory (A513). For the measurement process, the reflection accessory enables adjustment of the test angles, which cover a range of 13°–83° with a 1° angular resolution, and directs the polarized light from the FTIR to the detector (rT‐DLaTGS). Since our designed structure is stacked with a thick metal, resulting in negligible transmittance, the spectral angular emissivity of our fabricated structure can be calculated by e(λ,θ)=α(λ,θ)=1−R(λ,θ), where R(λ,θ) represents the measured spectral angular reflectivity.

### 4.3 Thickness Measurements

The thicknesses of each layer in the multilayer structure are measured by ellipsometry (Semilab SE‐2000).

### 4.4 Numerical Simulation

The reflectivity and absorptivity/emissivity spectra are simulated using the transfer matrix method. Electrical simulations are performed using the Lumerical's STACK optical solver. The Bayesian optimization (BO) process is implemented in Python.

### 4.5 Infrared Experiment

Infrared images and apparent temperatures are measured using an infrared camera (Guide PS 400) with a working wavelength range of 7.5–14 μm.

## Author Contributions

**Erwei Gui:** conceptualization, investigation, writing – original draft, methodology. **Guangji Lian:** software, methodology. **Shenghao Jin:** writing – review and editing, validation. **Jiahao Zhou:** investigation. **Shuai Gong:** investigation. **Changying Zhao:** funding acquisition. **Boxiang Wang:** conceptualization, methodology, funding acquisition, supervision, writing – review and editing, validation.

## Conflicts of Interest

The authors declare no conflicts of interest.

## Supporting information


Supporting Information S1

## Data Availability

The data that support the findings of this study are available from the corresponding author upon reasonable request.
