# Supplementary material for: Bayesian Optimized High‐Figure‐of‐Merit Broadband Directional Thermal Emitters
Source: Nanophotonics. 2026 Jan 14;15(1):e70003. doi: 10.1002/nap2.70003 (PMC12965028; doi:10.1002/nap2.70003)
Supplement: Supplementary file 1 — Supporting Information S1 [file NAP2-15-e70003-s001.docx]

Supporting Information

**Bayesian optimized high-figure-of-merit broadband directional thermal emitters**

Erwei Gui^1, 2, 3^, Guangji Lian^2, 3^, Shenghao Jin^2, 3^, Jiahao Zhou^1^, Shuai Gong^1^, Changying Zhao^1^, Boxiang Wang^2, 3, *^

Erwei Gui, Jiahao Zhou, Shuai Gong, Changying Zhao

^1^Institute of Engineering Thermophysics, School of Mechanical Engineering, Shanghai Jiao Tong University, Shanghai 200240, China

Erwei Gui, Guangji Lian, Shenghao Jin, Boxiang Wang

^2^2020 X-Lab, Shanghai Institute of Microsystem and Information Technology, Chinese Academy of Sciences, Shanghai, 200050, China

^3^State Key Laboratory of Transducer Technology, Shanghai Institute of Microsystem and Information Technology, Chinese Academy of Sciences, Shanghai, 200050, China

E-mail: [wangboxiang@mail.sim.ac.cn](mailto:wangboxiang@mail.sim.ac.cn) (Boxiang Wang)

Keywords: epsilon-near-zero (ENZ), directional thermal emission, Bayesian optimization, infrared deception

## Section S1. Transfer matrix method

For multilayer thin films, the reflectance and transmittance spectra are calculated using the transfer matrix method (TMM)^[1]^. For a multilayer film structure composed of K materials (layers 0, 1, 2, …, K-1), where layers 0 and K-1 represent the incident medium and exit medium, respectively, the reflection coefficient $r_{k,k+1}$ and transmission coefficient$t_{k,k+1}$ at the interface between the $k$th and $\left( k+1 \right)$th layers can be determined by

$$\begin{aligned} \begin{matrix} r_{s}=\frac{n_{k}\cos\theta_{k}-n_{k+1}\cos\theta_{k+1}}{n_{k}\cos\theta_{k}+n_{k+1}\cos\theta_{k+1}}, \\ r_{p}=\frac{n_{k+1}\cos\theta_{k}-n_{k}\cos\theta_{k+1}}{n_{k+1}\cos\theta_{k}+n_{k}\cos\theta_{k+1}}, \end{matrix}\#\left( S S\mathrm{EQ}公式 1 \right) \end{aligned}$$

$$\begin{aligned} \begin{matrix} t_{s}=\frac{2n_{k}\cos\theta_{k}}{n_{k}\cos\theta_{k}+n_{k+1}\cos\theta_{k+1}}, \\ t_{p}=\frac{2n_{k}\cos\theta_{k}}{n_{k+1}\cos\theta_{k}+n_{k}\cos\theta_{k+1}}, \end{matrix}\#\left( S S\mathrm{EQ}公式 2 \right) \end{aligned}$$

where $n$ is the complex refractive index,$\theta_{k}$ refers to the incident angle of $k$th layer. The transfer matrix of the interface between the $k$th and $(k+1)$th layers can then be described as

$$\begin{aligned} I_{k}\equiv\left( \begin{matrix} e^{-i\delta_{k}} & 0 \\ 0 & e^{i\delta_{k}} \end{matrix} \right)\left( \begin{matrix} 1 & r_{k,k+1} \\ r_{k,k+1} & 1 \end{matrix} \right)\frac{1}{t_{k,k+1}},\#\left( S S\mathrm{EQ}公式 3 \right) \end{aligned}$$

where $\delta_{k}$characterizes the phase that comes from passing through layer k, and it can be described by

$\begin{aligned} \delta_{k}=d_{k}\frac{2\pi n_{k}}{\lambda}\cos\theta_{k},\#\left( S S\mathrm{EQ}公式 4 \right) \end{aligned}$where $d_{k}$is the thickness of kth layer. Then, the total reflection coefficient $r$ and total transmission coefficient $t$ of the entire multilayer structure can be determined from the total transfer matrix $I_{K}$

$$\begin{aligned} I_{K}=\frac{1}{t_{0}}\left( \begin{matrix} 1 & r_{0} \\ r_{0} & 1 \end{matrix} \right)I_{1}I_{2}\cdots I_{K-2},\#\left( S S\mathrm{EQ}公式 5 \right) \end{aligned}$$

$$\begin{aligned} \left( \begin{aligned} 1 \\ r \end{aligned} \right)=\left( \begin{matrix} I_{K}(1,1) & I_{K}(1,2) \\ I_{K}(2,1) & I_{K}(2,2) \end{matrix} \right)\left( \begin{aligned} t \\ 0 \end{aligned} \right),\#\left( S S\mathrm{EQ}公式 6 \right) \end{aligned}$$

Finally, the spectral reflectance and transmittance of the structure can be described by

$$\begin{aligned} R=\left| r \right|^{2},\#\left( S S\mathrm{EQ}公式 7 \right) \end{aligned}$$

$$\begin{aligned} \begin{matrix} T_{s}=\left| t \right|^{2}\frac{\mathrm{Re}\left( n_{K-1}\cos\theta_{K-1} \right)}{\mathrm{Re}\left( n_{0}\cos\theta_{0} \right)}, \\ T_{p}=\left| t \right|^{2}\frac{\mathrm{Re}\left( n_{K-1}\cos\theta_{K-1}^{*} \right)}{\mathrm{Re}\left( n_{0}\cos\theta_{0}^{*} \right)}, \end{matrix}\#\left( S S\mathrm{EQ}公式 8 \right) \end{aligned}$$

## Section S2. Admittance calculation

Admittance is defined as the ratio of the magnetic field to the electric field, and the effective admittance of the multilayer structure is calculated as^[2]^

$$\begin{aligned} \left[ \begin{aligned} E_{Z} \\ H_{Z} \end{aligned} \right]=\prod_{z=1}^{Z} \left[ \begin{matrix} \cos\delta_{z} & -\frac{\mathrm{isin} \delta_{z}}{\eta_{z}} \\ -i\eta_{z}\sin\delta_{z} & \cos\delta_{z} \end{matrix} \right]\left[ \begin{aligned} 1 \\ \eta_{\mathrm{sub}} \end{aligned} \right],\#\left( \text{S}\text{}\text{ SEQ }\text{公式}\text{ }\text{}\text{9}\text{} \right) \end{aligned}$$

where $Z$ is the number of layers. $\delta$ and $\eta$ are the phase shift and admittance, respectively. The admittance for p-polarized light is calculated as $\eta=\text{N}/{\text{cos}\text{ }\text{(}\theta)}$, where $N=n+ik$ represents the complex refractive index of the material and $\theta$ refers to the incidence angle. $\delta$ is given by ${2\pi\text{N}d\cos\theta}/\lambda$, where $d$ is the thickness of the layer. $\eta_{\text{sub}}$ is the admittance of the substrate, and the effective admittance of the multilayer film structure can be calculated as $\eta_{e}={H_{Z}}/{E_{Z}}$.

## Section S3. FP mode of the Ge layer

To suppress the small-angle absorption induced by ENP resonance of ENZ materials, the Ge layer must satisfy the FP resonance condition: *d*=$\lambda/(4n_{\mathrm{Ge}})$. When the Ge layer thickness matches this condition, the structure exhibits high reflectance at normal incidence over a broadband range (centered around the designed wavelength) due to constructive interference of incident light, effectively suppressing small-angle absorption. Moreover, the coupling between the FP mode and the Brewster effect of the Ge layer results in high reflectance at small angles and high transmittance at large angles across a broad spectral range.

## Section S4. Color difference calculation

In order to achieve visible camouflage, we design the thickness of the Si layer sputtered on the Ag layer via color difference calculation, ensuring that the visible color of the designed covers matches that of the BDTE sample. The CIE 1931 color coordinate system based on the CIE$XYZ$ coordinate space is used to calculate the color difference$\Delta E$ between the BDTE sample and the cover. The tristimulus values $X$, $Y$, and $Z$ of color can be calculated using CIE color-matching functions $\bar{x}\left( \lambda\right)$, $\bar{y}\left( \lambda\right)$, $\bar{z}\left( \lambda\right)$, and reflection spectrum of the BDTE sample $R\left( \lambda\right)$ as^[3]^

$$\begin{aligned} X=k\int_{\lambda_{b}}^{\lambda_{a}} R\left( \lambda\right)D65\left( \lambda\right)\bar{x}\left( \lambda\right)d\lambda,\#\left( \text{S}\text{}\text{ SEQ }\text{公式}\text{ }\text{}\text{10}\text{} \right) \end{aligned}$$

$$\begin{aligned} Y=k\int_{\lambda_{b}}^{\lambda_{a}} R\left( \lambda\right)D65\left( \lambda\right)\bar{y}\left( \lambda\right)d\lambda,\#\left( \text{S}\text{}\text{ SEQ }\text{公式}\text{ }\text{}\text{11}\text{} \right) \end{aligned}$$

$$\begin{aligned} Z=k\int_{\lambda_{b}}^{\lambda_{a}} R\left( \lambda\right)D65\left( \lambda\right)\bar{z}\left( \lambda\right)d\lambda,\#\left( \text{S}\text{}\text{ SEQ }\text{公式}\text{ }\text{}\text{12}\text{} \right) \end{aligned}$$

where $D65\left( \lambda\right)$ is spectral power distribution of CIE Illuminant D65. $\lambda_{a}$ and $\lambda_{b}$ refer to the maximum and minimum wavelengths for integration, and $\lambda_{a}=\text{0.8 }\text{μm}$, $\lambda_{b}=\text{0.36 }\text{μm}$.$k$ is the normalization parameter and $k=100/\int_{\lambda_{b}}^{\lambda_{a}} D65\left( \lambda\right)\bar{y}\left( \lambda\right)d\lambda$. Then, the CIE$XYZ$ coordinate space must transform to CIE$L^{*}a^{*}b^{*}$ coordinate space to calculate the color difference: ^[4]^

$$\begin{aligned} L^{*}=116f\left( \frac{Y}{Y_{n}} \right)-16,\#\left( \text{S}\text{}\text{ SEQ }\text{公式}\text{ }\text{}\text{13}\text{} \right) \end{aligned}$$

$$\begin{aligned} a^{*}=500\left( f\left( \frac{X}{X_{n}} \right)-f\left( \frac{Y}{Y_{n}} \right) \right),\#\left( \text{S}\text{}\text{ SEQ }\text{公式}\text{ }\text{}\text{14}\text{} \right) \end{aligned}$$

$$\begin{aligned} b^{*}=200\left( f\left( \frac{Y}{Y_{n}} \right)-f\left( \frac{Z}{Z_{n}} \right) \right),\#\left( \text{S}\text{}\text{ SEQ }\text{公式}\text{ }\text{}\text{15}\text{} \right) \end{aligned}$$

where $X_{n}=95.047$, $Y_{n}=100$, $Z_{n}=108.883$ are the CIE XYZ reference tristimulus values (for CIE Illuminant D65) and the function $f\left( x \right)$ is

$$\begin{aligned} f\left( x \right)=\left\{ \begin{aligned} x^{\frac{1}{3}}, x>\left( \frac{6}{29} \right)^{3} \\ \frac{x}{3\times\left( \frac{6}{29} \right)^{2}}+\frac{4}{29}, x\leq\left( \frac{6}{29} \right)^{3} \end{aligned} \right.\#\left( \text{S}\text{}\text{ SEQ }\text{公式}\text{ }\text{}\text{16}\text{} \right) \end{aligned}$$

Finally, the color difference ∆E is determined by the differences in $\Delta L^{*}$ , $\Delta a^{*}$, and $\Delta b^{*}$ between the BDTE sample and the designed covers:

$$\begin{aligned} \Delta E=\sqrt{\left( \Delta L^{*} \right)^{2}+\left( \Delta a^{*} \right)^{2}+\left( \Delta b^{*} \right)^{2}}\#\left( \text{S}\text{}\text{ SEQ }\text{公式}\text{ }\text{}\text{17}\text{} \right) \end{aligned}$$

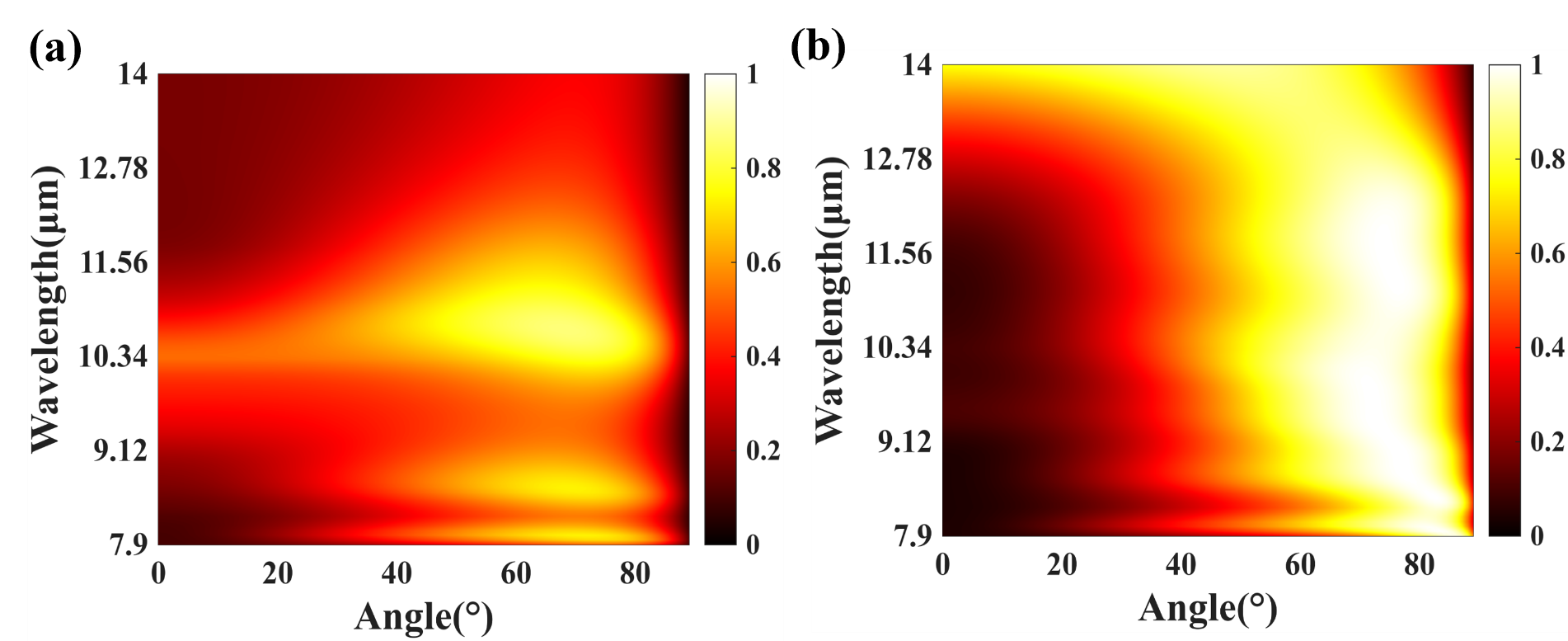


**Figure S1.** a) Calculated angle-resolved emissivity spectrum of the multilayer structure (TiO_2_/SiO/SiO_2_/Al_2_O_3_/Ag) under *p* polarization. The thicknesses of each ENZ layer are 337, 293, 88, and 229 nm, respectively (from top to bottom), which are determined by Equation (1) in the main text with an emission angle of 80$^{\circ}$. b) Calculated angle-resolved emissivity spectrum of the multilayer structure (TiO_2_/SiO/SiO_2_/Ge/Al_2_O_3_/Ag) under *p* polarization. The thicknesses of each ENZ layer are 337, 293, 88, and 229 nm, respectively (from top to bottom), and the Ge layer has a thickness of 661 nm, which is determined by the FP mode of the Ge layer at a wavelength of 11 μm.

Directly stacking the ENZ multilayer films (with the calculated thicknesses) on a metal results in undesired small-angle absorption due to TO resonance caused by the excessive total thickness of the multilayer structure. After adding the Ge layer, the directional performance is improved. However, the angular range of directional emission $\Delta\theta$ of this structure (Figure S1b) exceeds 52$^{\circ}$ (the angle range that satisfies$\bar{e}\left( \theta_{i} \right)>{\bar{e}_{max}}/2$) in the long wave-infrared (LWIR) range. Additionally, it shows almost no broadband directional emission effect in the non-ENZ wavelength band (12-14$\mu m$), which thus requires further optimization.


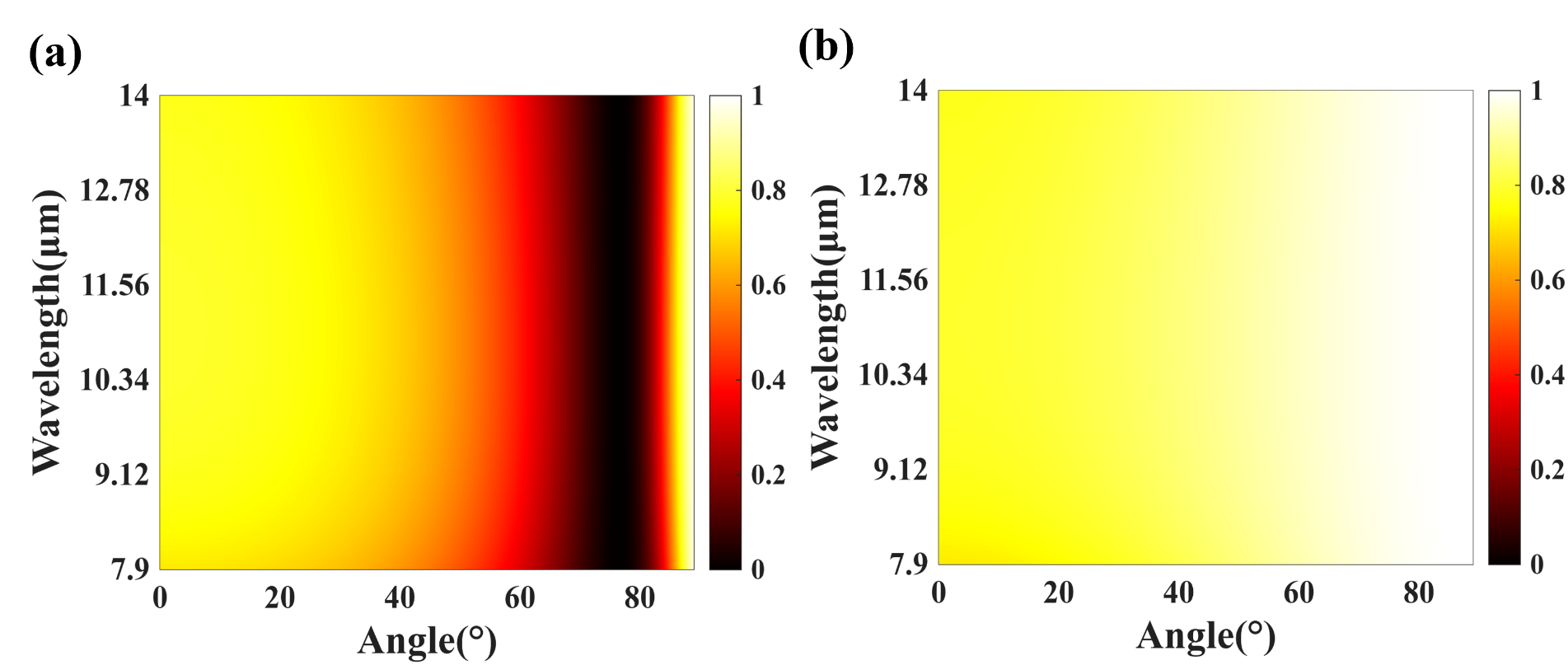


**Figure S2.** a) Calculated angle-resolved reflectivity spectrum under *p* polarization of the freestanding Ge layer with a thickness of 661 nm, which is determined by the FP mode at a wavelength of 11 $\mu m$. b) Calculated angle-resolved reflectivity spectrum under *s* polarization of the freestanding Ge layer with a thickness of 661 nm.


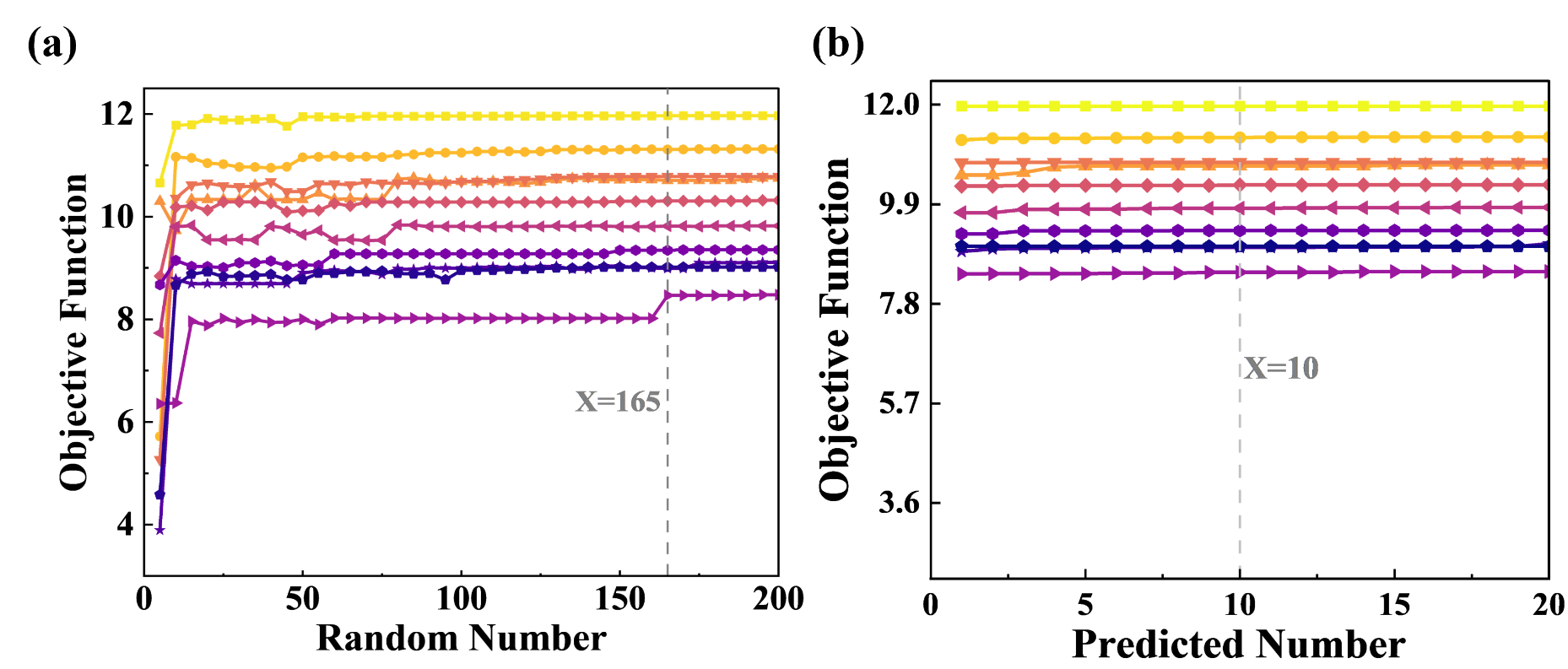


**Figure S3.** a) Histories of the objective function (OF) values for 10 predicted candidate structures, varying with the number of initial random candidate structures across 10 random groups in Process I. b) Histories of the OF values for 165 initial random candidate structures, varying with the number of predicted candidate structures across 10 random groups in Process I.


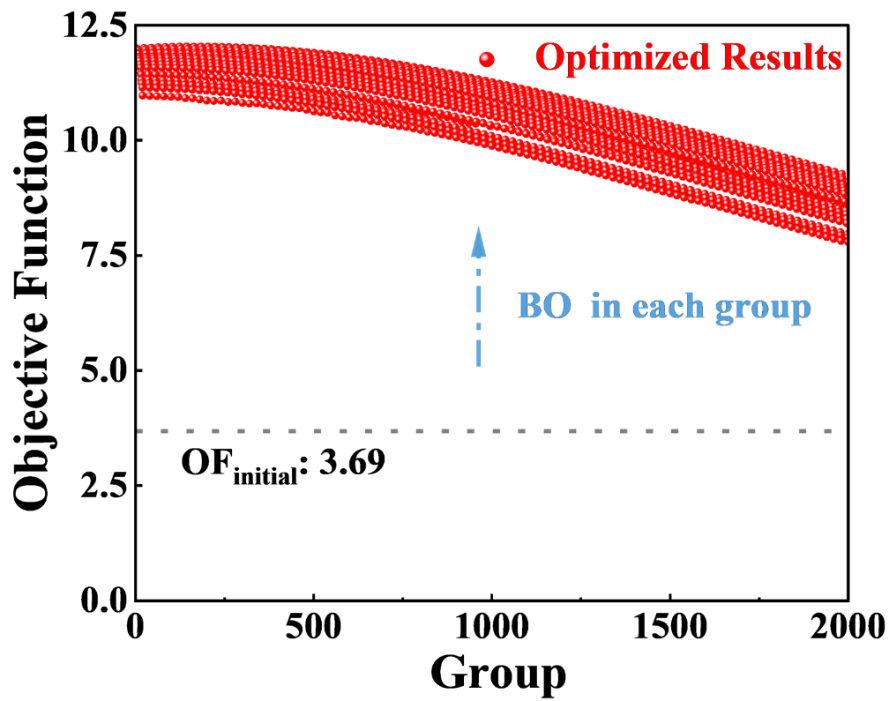


**Figure S4.** The optimized results of 2000 groups. The initial OF of the structure with arbitrarily selected initial integer thicknesses is 3.69.


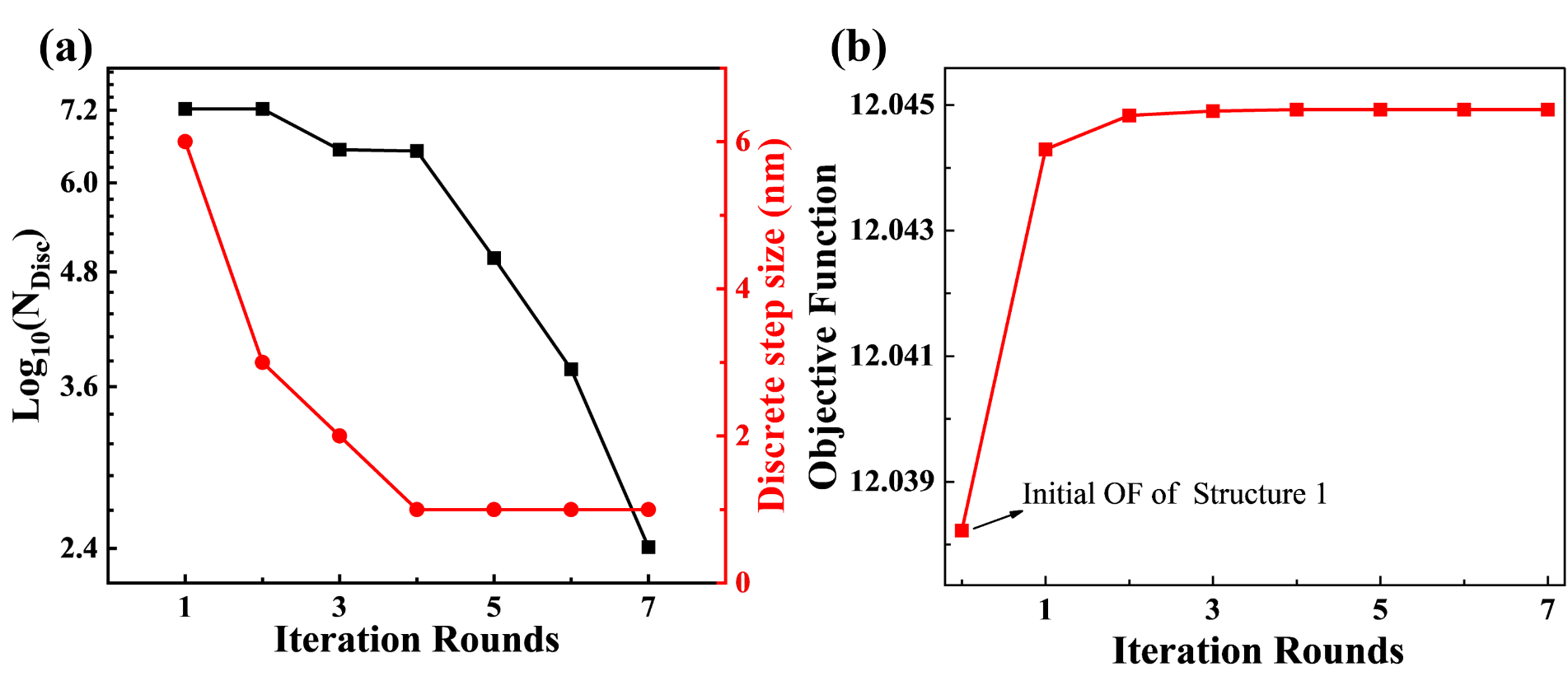


**Figure S5.** a) The number of initial candidate structures and discrete thickness step size vary with iteration rounds proceed in Process Ⅱ. b) The OF values of the optimized structures vary with iteration rounds proceed in Process Ⅱ.


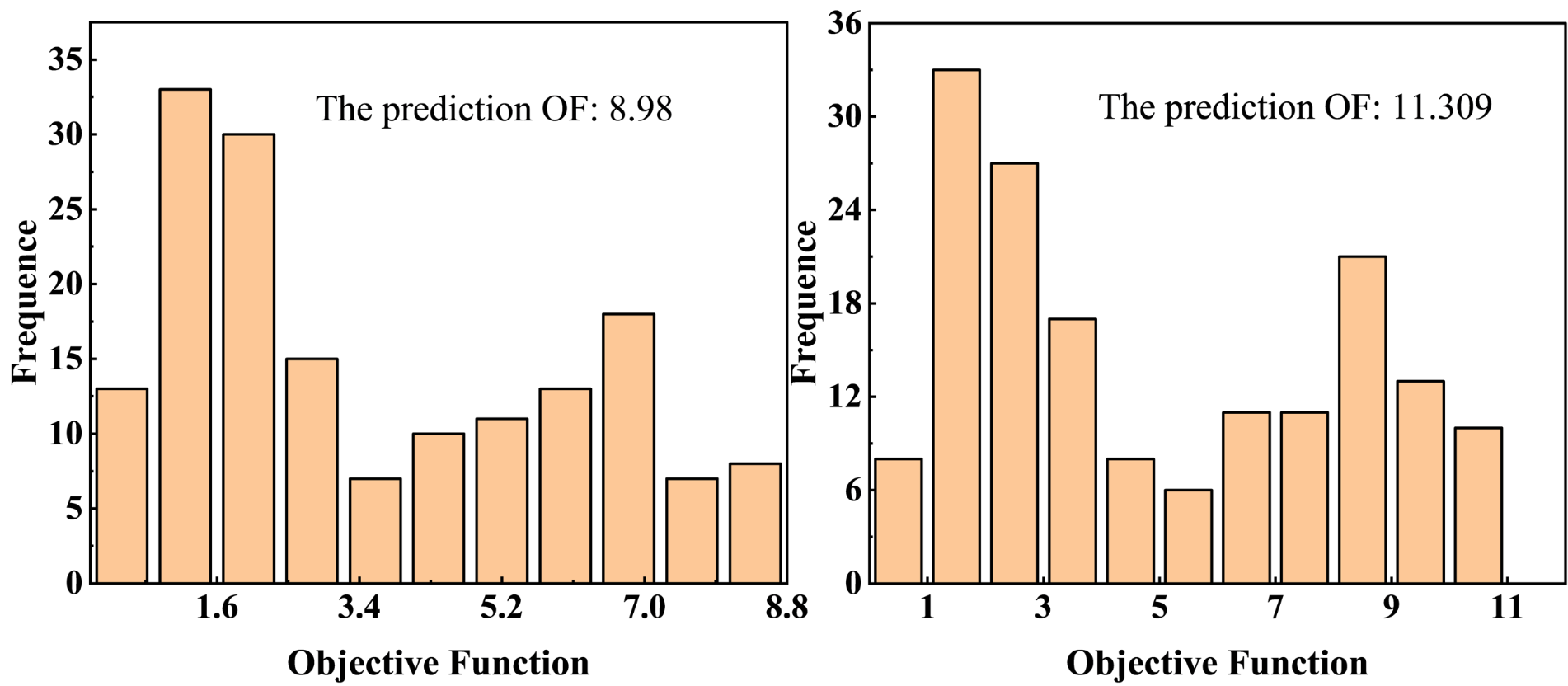


**Figure S6**. The frequency distribution of OF values for initial random candidate structures of 2 random groups in Process I. The OF value of the optimized structure in each group is superior to those of the initial random candidate structures.


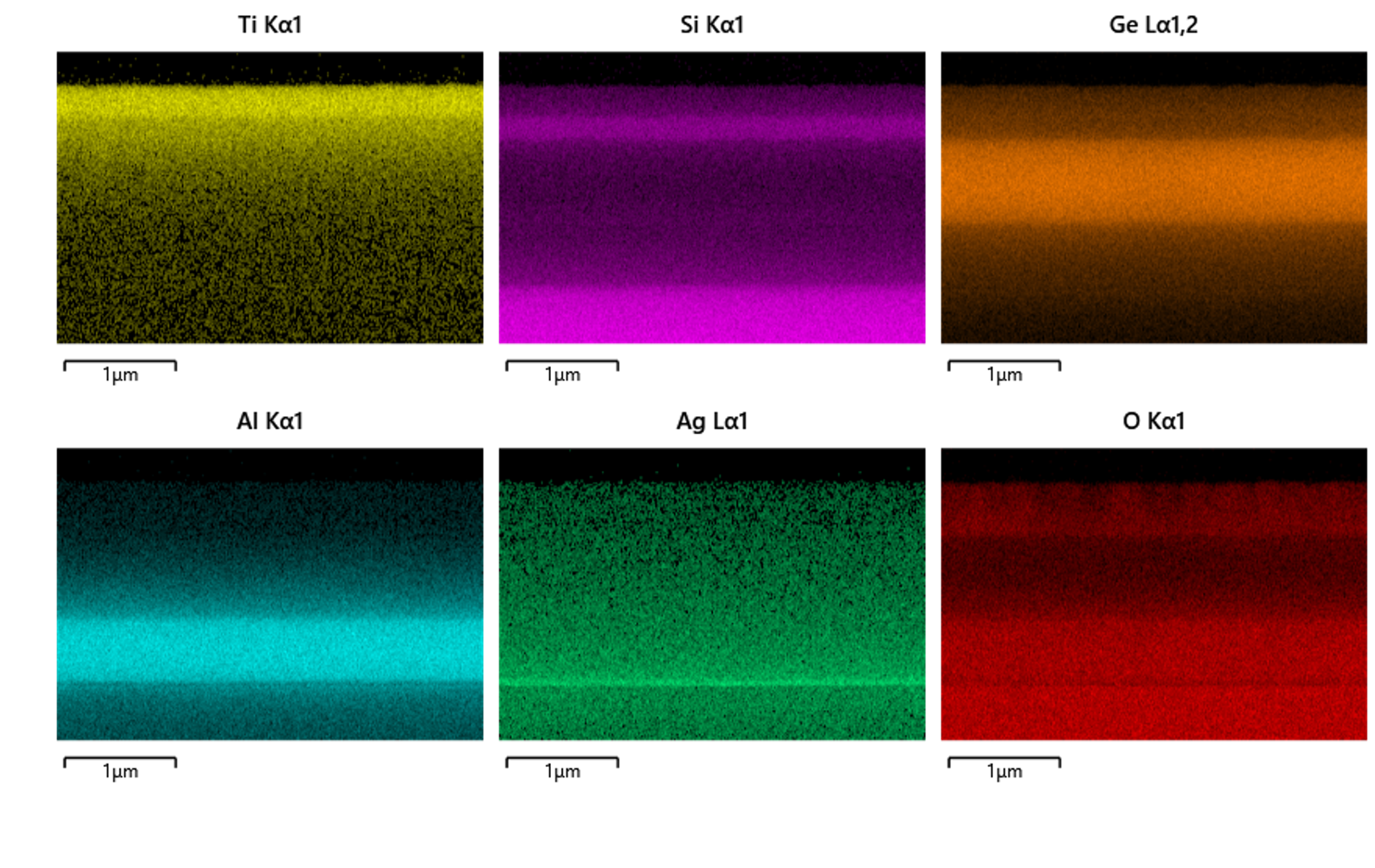


**Figure S7.** EDS images of each element in fabricated multilayer structure.


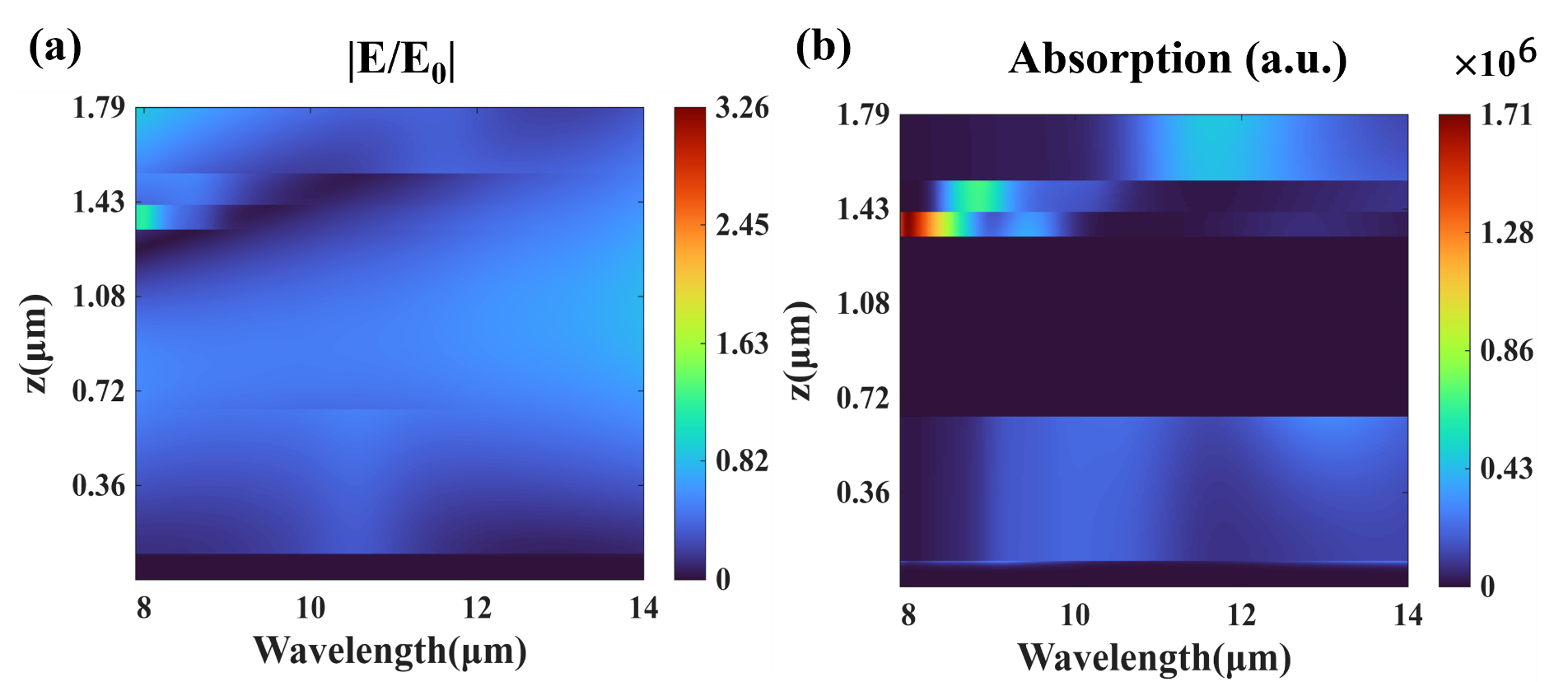


**Figure S8.** a) Calculated electric field distributions across the *z* direction at different wavelengths under *p* polarization at an incident angle of 10°. b) Calculated bulk absorption distributions in each layer at different wavelengths for *p* polarization at an incident angle of 80$^{\circ}$.


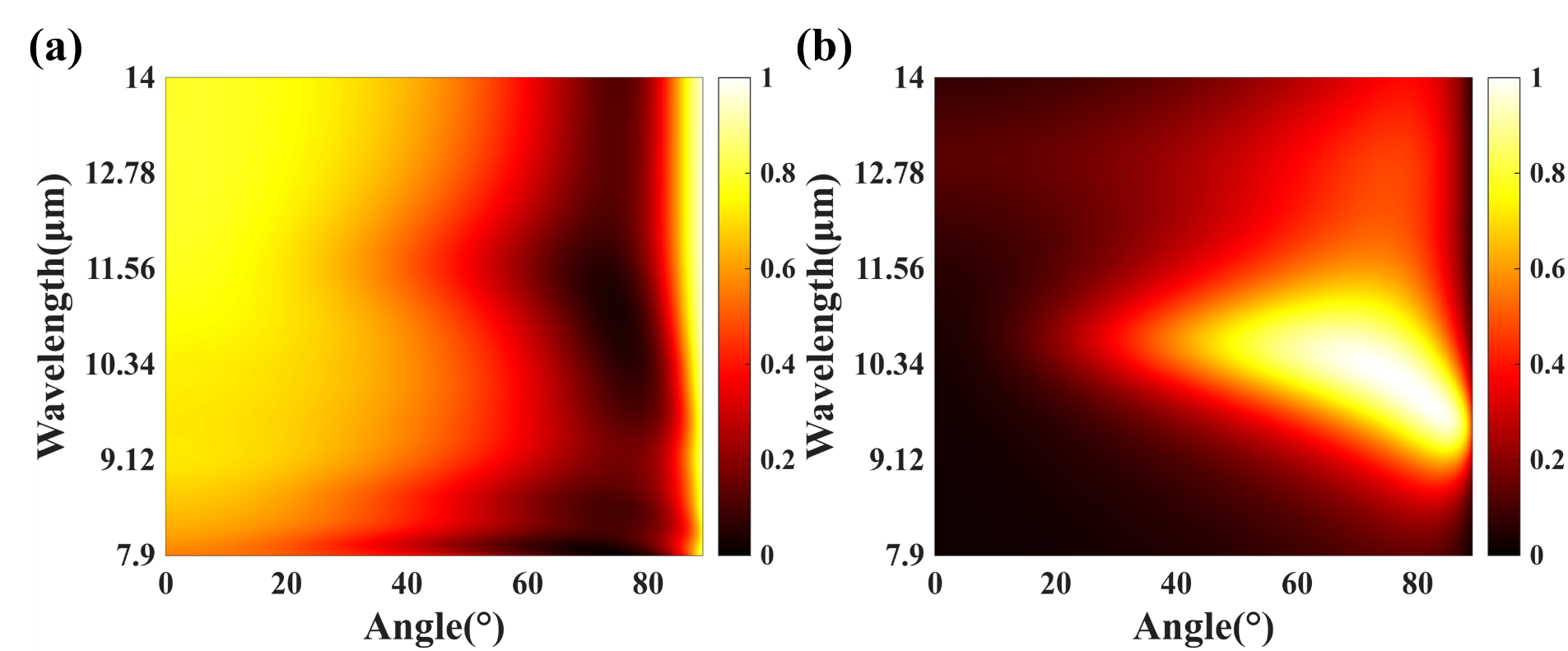


**Figure S9.** a) Calculated angle-resolved reflectivity spectrum of the upper three ENZ material layers after stacking with Ge at the bottom with optimized thickness under *p* polarization. b) Calculated angle-resolved reflectivity spectrum of the Al_2_O_3_ film with an optimized thickness (547 nm) stacked on the metal substrate under *p* polarization.


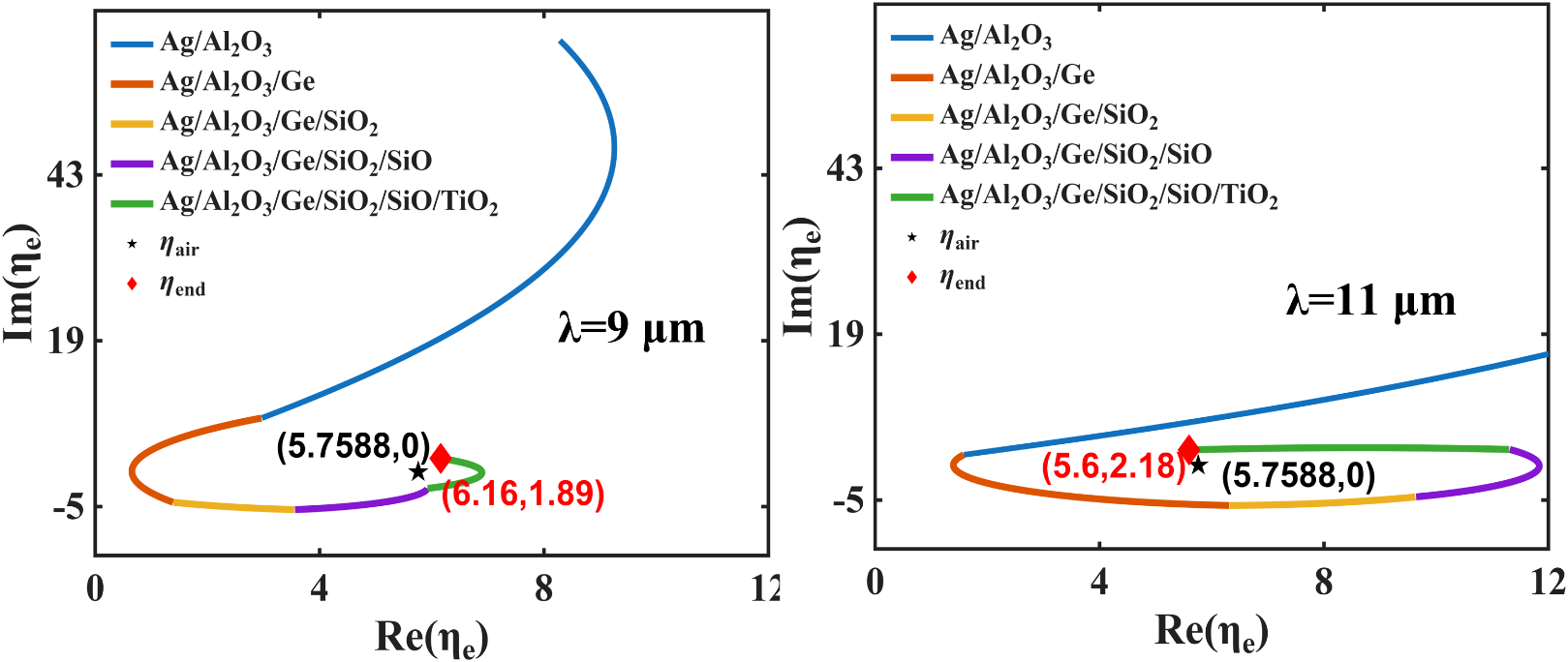


**Figure S10.** The admittance trajectories of the multilayer structure when stacked sequentially under *p* polarization and 80° incidence at different wavelengths (left: $\lambda=$9 $\mu m$, right: $\lambda=$11$\mu m$).


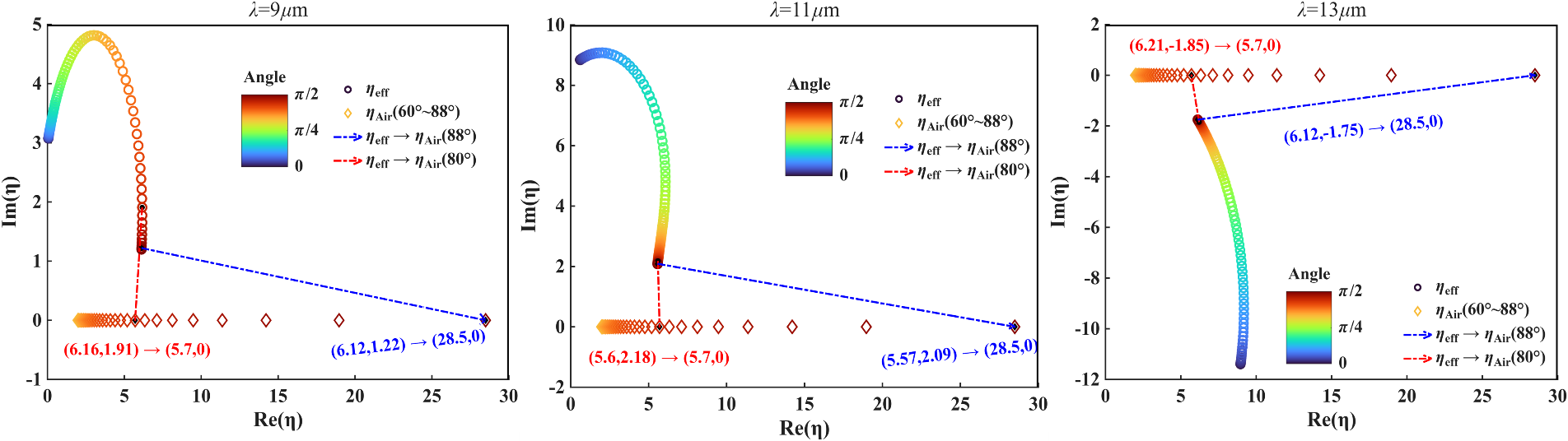


**Figure S11.** The admittance trajectories of the designed multilayer structure as a function of incident angle under *p* polarization at different wavelengths (left: $\lambda=$9 $\mu m$, middle: $\lambda=$11$\mu m$, right: $\lambda=$13$\mu m$).


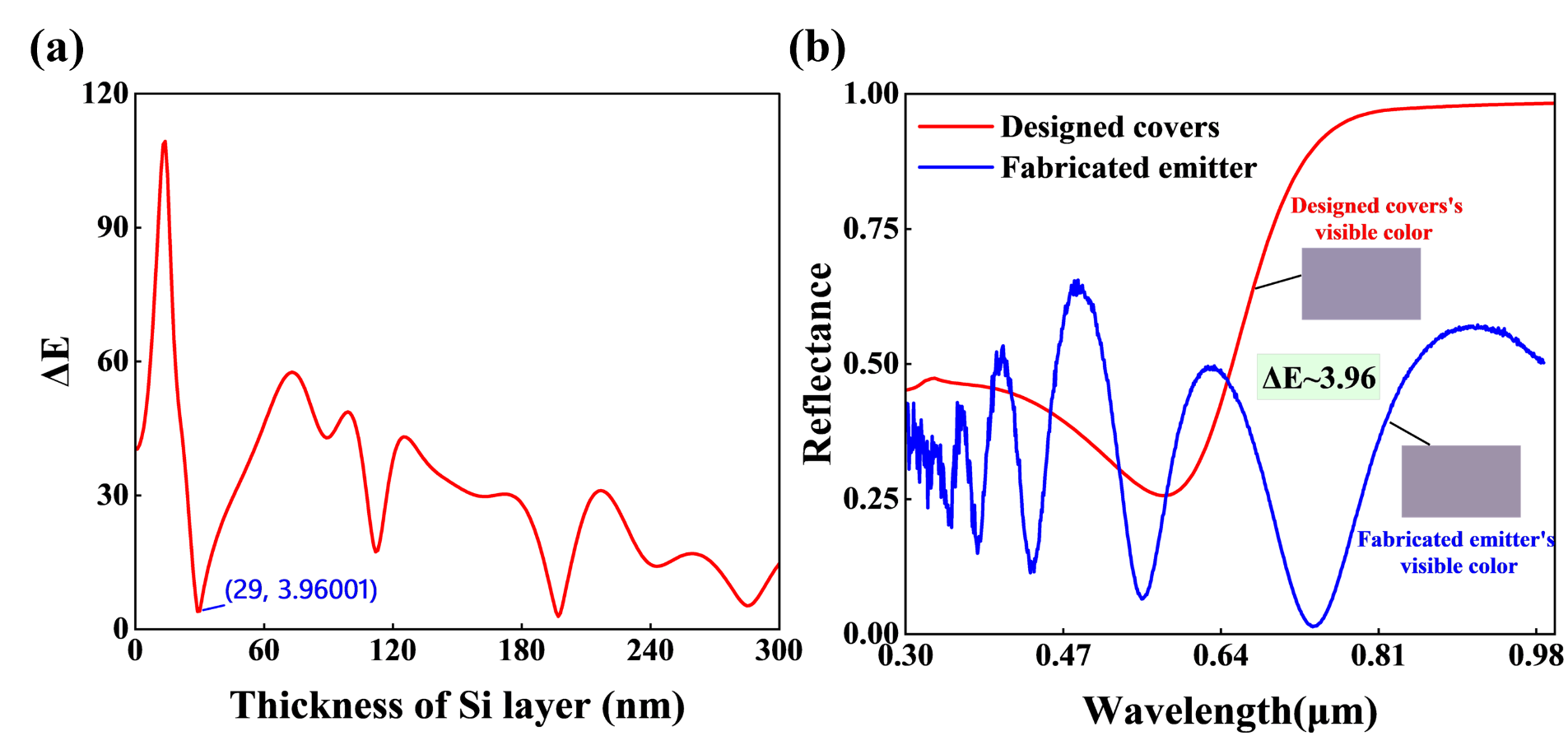


**Figure S12.** a) Calculated color difference ∆E between the designed covers and the fabricated emitter varying with the thickness of Si layer. b) Reflectivity spectra and visible colors of the fabricated BDTE sample (blue line) and the designed covers (red line) within the wavelength range of 0.3-1 μm under normal incidence.

For the design of the covers, a 60 nm-thick Ag is deposited on the hollowed-out quartz to achieve infrared (IR) low emission. To realize the visible camouflage, the visible color of the covers should match that of the fabricated emitter closely. However, the Ag/Quartz structure exhibits high reflectance in the visible range, which renders the covers easily identified when they cover the BDTE sample. To address this issue, we select the Si material (lossless in the LWIR band) and deposit it on the Ag layer to tune the color of the covers without increasing the IR emission. From the color calculation (Section S4) based on the sample’s reflectance spectrum, the three CIE 1976 $L^{*}a^{*}b^{*}$ coordinate values of the fabricated emitter are obtained as: $L_{sam}^{*}$ = 62.2151, $a_{sam}^{*}$= 9.0834, and $b_{sam}^{*}$= -10.2344. As shown in Figure S12a, the color difference ∆E between the designed covers and the fabricated emitter is calculated, which varies with the thickness of the Si layer. When the thickness of the Si layer is set to 29 nm, the color difference between the designed covers and the fabricated BDTE sample is 3.96 (less than 5), exhibiting great potential for visible concealment. The reflectivity spectra and visible color images of the fabricated BDTE sample (blue line) and the designed covers (red line) are shown in Figure S12b. By setting the thickness of the Si layer to 29 nm, the visible colors of the covers and the emitter are highly similar, which are difficult to distinguish, demonstrating good camouflage performance in the visible range. Meanwhile, the IR emission of the covers remains low, which can be verified from the IR images (Figure 4e in the main text), thus not impacting the IR encryption and deception performance.


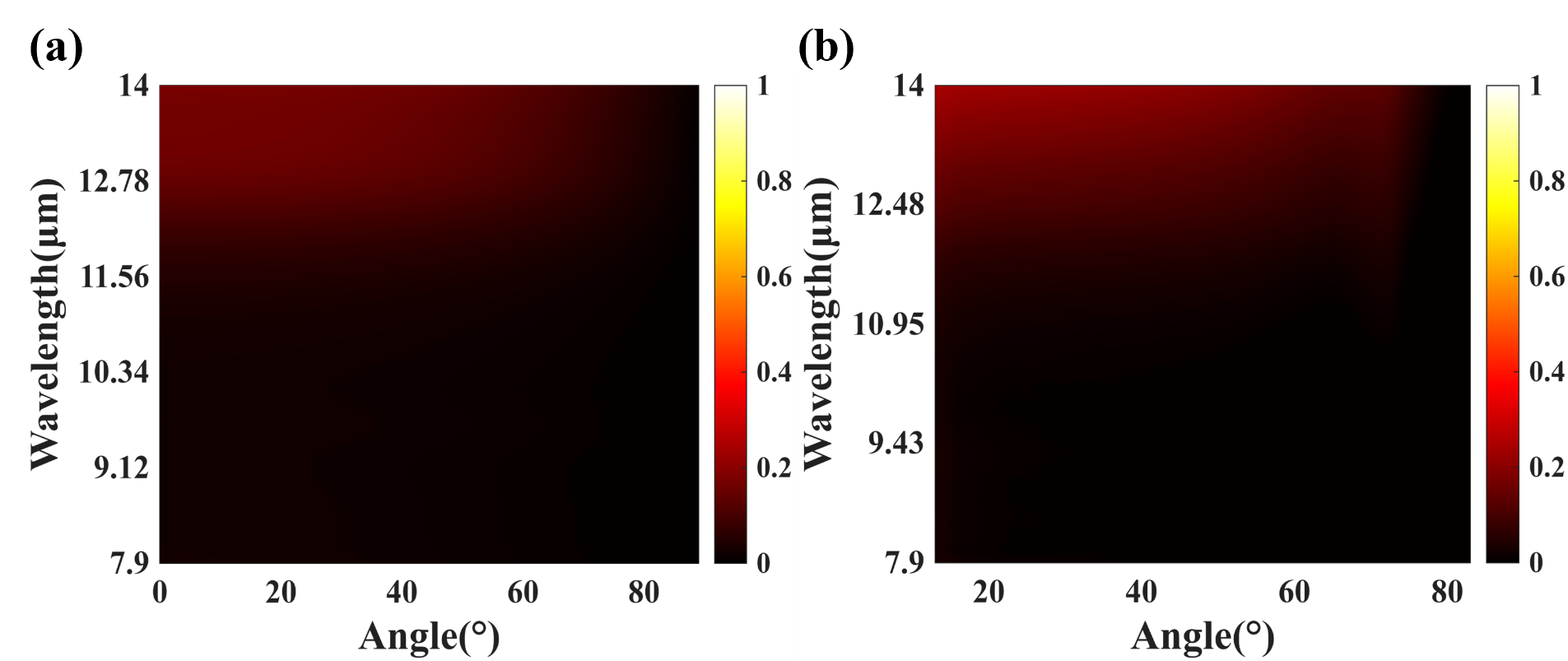


**Figure S13.** a) Calculated angle-resolved emissivity spectrum of designed multilayer emitter under *s* polarization. b) Measured angle-resolved emissivity spectrum of the fabricated multilayer emitter under *s* polarization.


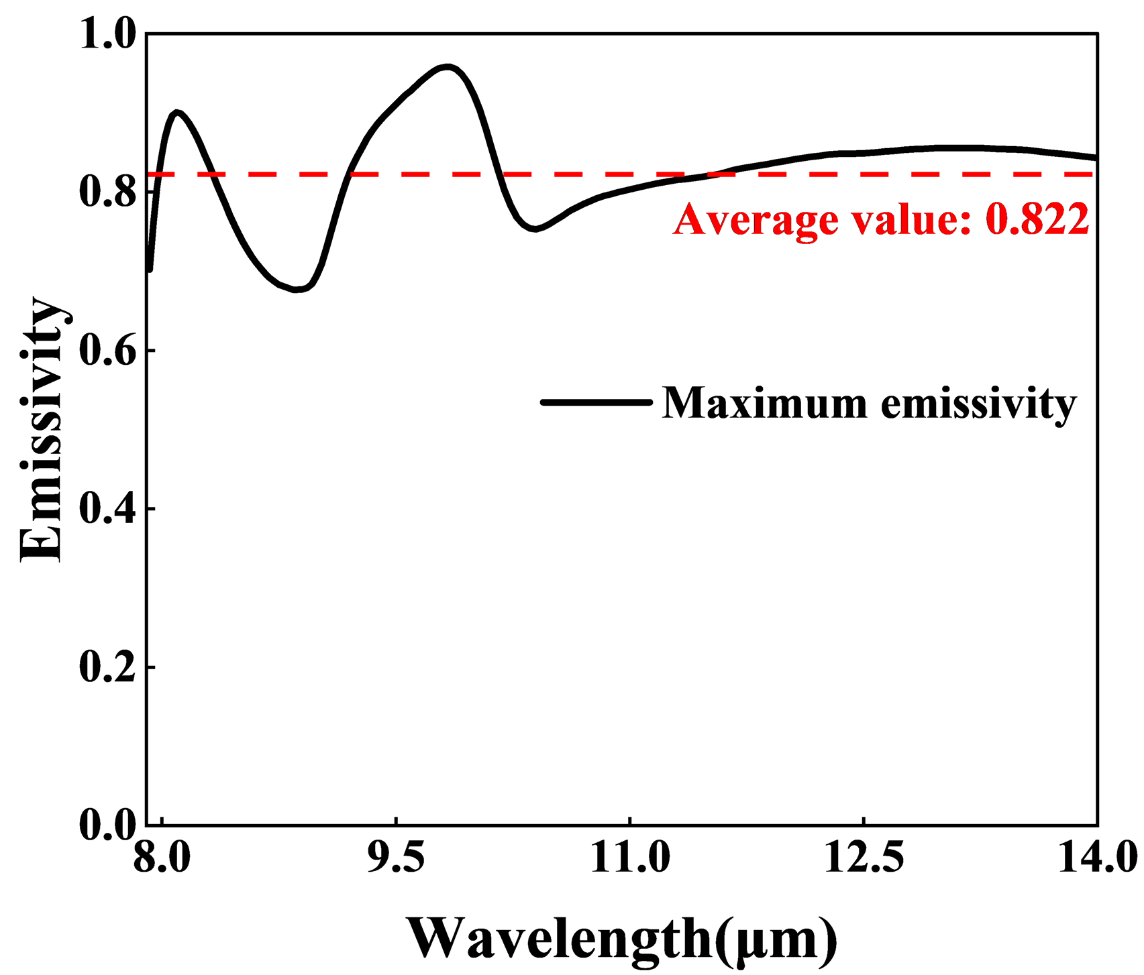


**Figure S14.** Measured maximum spectral emissivity of the fabricated sample within the wavelength range of 7.9-14 μm. The emission angles corresponding to the maximum spectral emissivity vary with wavelength but all fall in the desired angle range of 60°-83°.


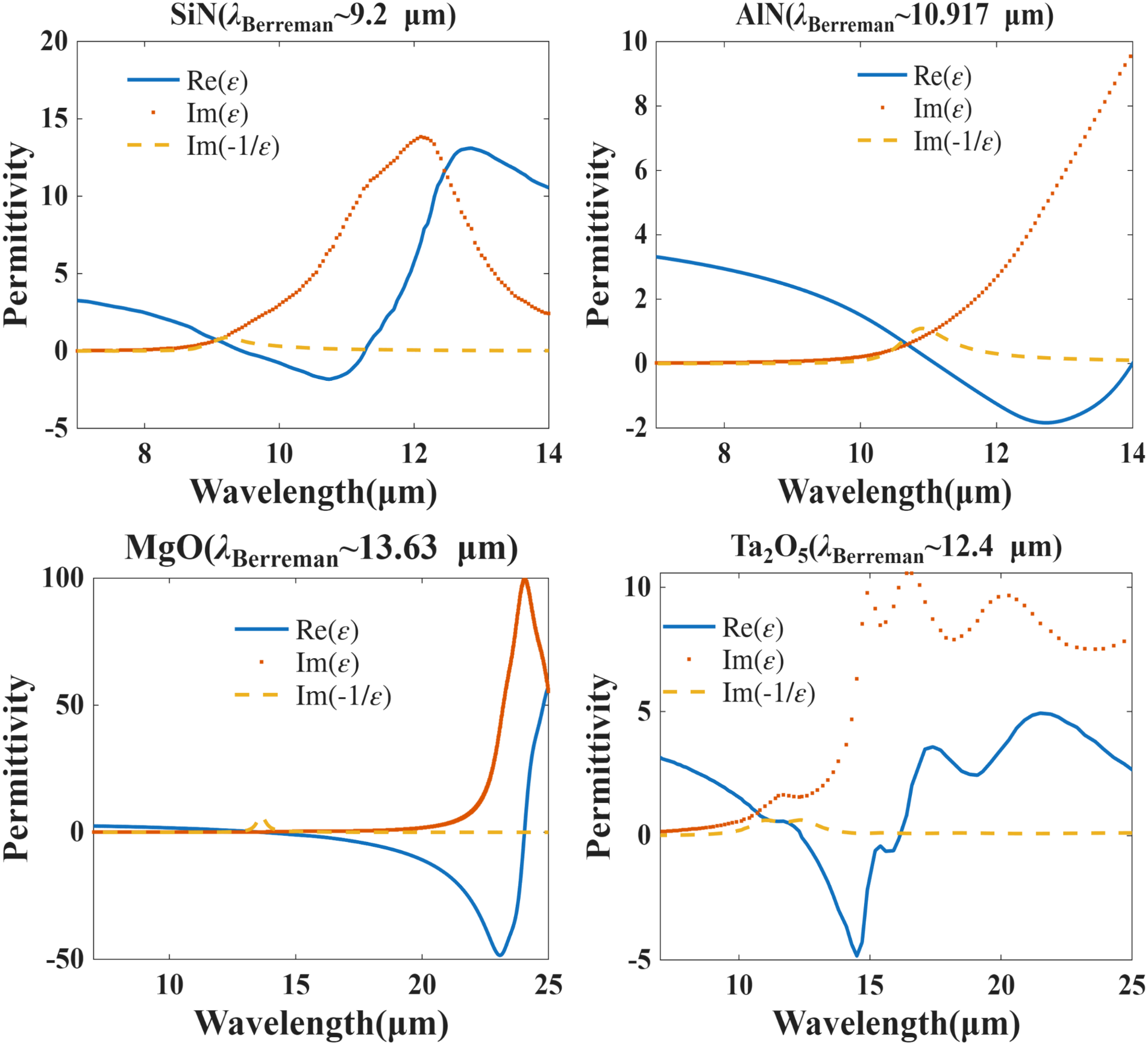


**Figure S15.** The permittivities of other ENZ materials,^[5–7]^ which exhibit great potential for broadening the BDTE wavelength range. Due to the ENP resonance, SiN and AlN materials demonstrate strong absorption capability near 12 μm and 14 μm, respectively. MgO and Ta_2_O_5_ also exhibit intense absorption near 24 μm and within the range of 15-25 $\mu m$, respectively.


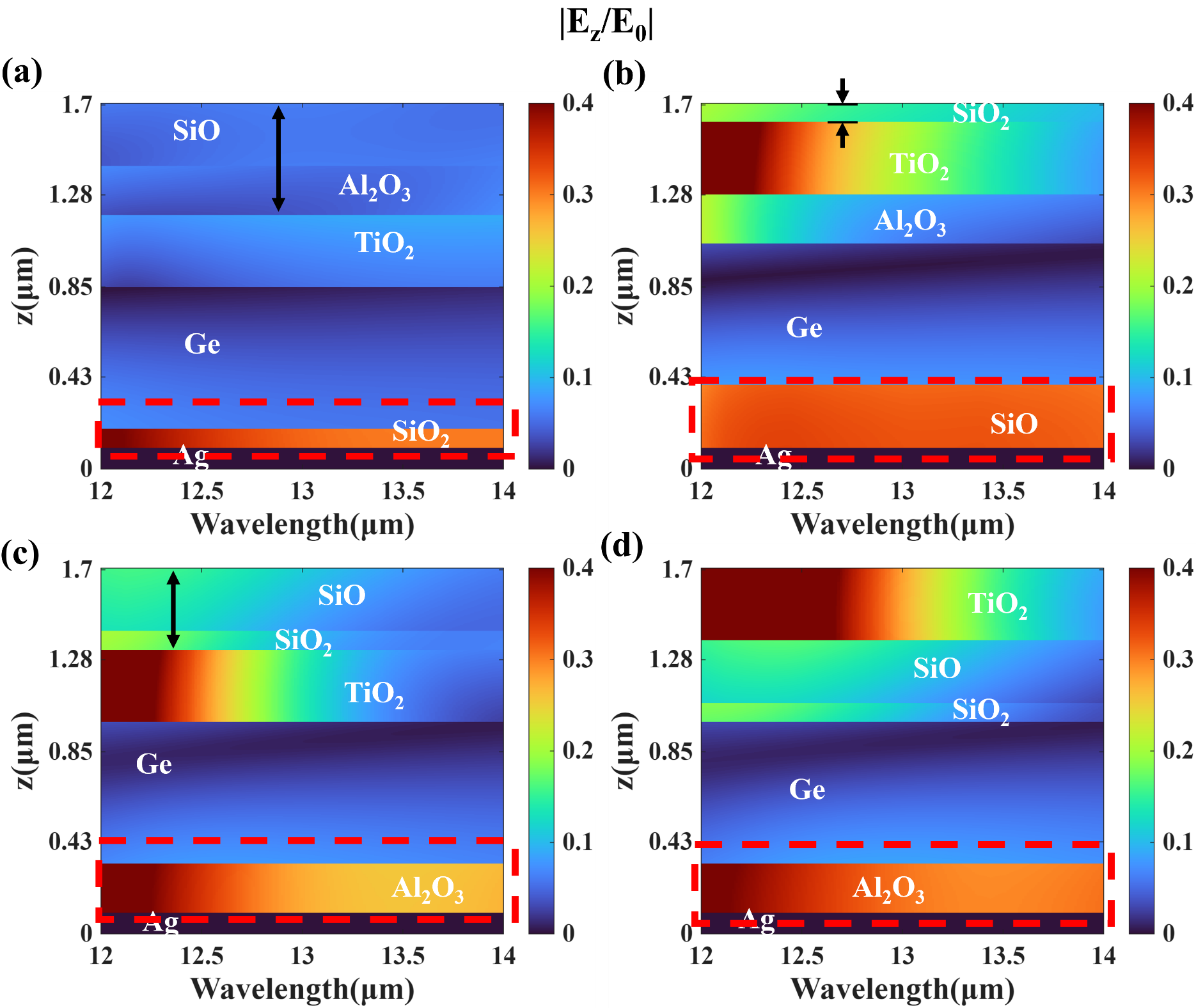


**Figure S16.** Calculated longitudinal electric field distributions for different stacking sequences (from top to bottom: a) SiO, Al_2_O_3_, TiO_2_, Ge, SiO_2_; b) SiO_2_, TiO_2_, Al_2_O_3_, Ge, SiO; c) SiO, SiO_2_, TiO_2_, Ge, Al_2_O_3_; d) TiO_2_, SiO, SiO_2_, Ge, Al_2_O_3_) across the *z* direction in the 12-14 μm range under p polarization at an incident angle of 80°. The thicknesses of TiO_2_, SiO, SiO_2_ and Al_2_O_3_ are 337, 293, 88, and 229 nm, respectively, which are calculated via Equation (1) in the main text for an emission angle of 80°. Moreover, the Ge layer has a thickness of 661 nm, which is determined by the FP mode at a wavelength of 11 μm.

Since the selected materials are unable to support Berreman mode in the non-ENZ wavelength range, achieving excellent large-angle absorption performance in the 12-14 μm range is more essential for the arrangement design of our structure. For large-angle incidence, owing to the excellent absorption capability (a large imaginary part of the permittivity) of TiO_2_ and Al_2_O_3_ within the 12-14 μm range, the strong enhancement of the longitudinal electric field in these layers enables the structure to induce strong absorption more efficiently. As shown in **Figure S16**, through multiple rounds of stacking sequence verification, the material layer between the metal substrate and Ge layer exhibits a more strongly enhanced and confined electric field than other layers (**Figure S16**a, **S16**b and **S16**c). Moreover, when the TiO_2_ layer is close to the incident medium (air), this layer displays a more strongly enhanced electric field that is conducive to achieving enhanced absorption. Therefore, the Al_2_O_3_ layer and the TiO_2_ layer are positioned between the Ge layer and metal layer and on top of the structure respectively to enable a greater electric field confinement. Additionally, since SiO_2_ and SiO materials exhibits negligible absorption capability (Im(ε)~0) in 12-14 μm range, no absorption is induced even in the presence of an electric field, thus, the SiO_2_ and SiO layers are consequently positioned on the Ge layer. The final configuration is TiO_2_/SiO/SiO_2_/Ge/Al_2_O_3_, which demonstrates the desired electric field confinement to realize the excellent large-angle absorption covering the 12-14 $\mu m$ range (**Figure S16**d).


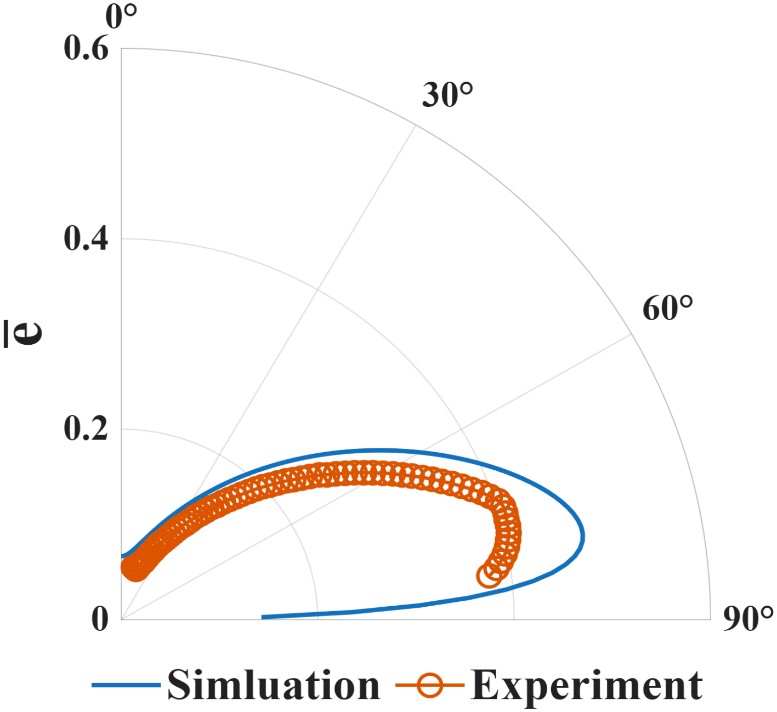


**Figure S17.** The simulated and experimental non-polarized average emissivity of designed BDTE structure varying with emission angle in the 7.9-14 $\mu m$ range. The simulated non-polarized average emissivity exhibits $\bar{e}_{\max}\sim$0.4785 (at 79$^{\circ}$) and $\bar{e}_{\min}\sim$0.0665 (at 0$^{\circ}$), respectively, which correspond to an emissivity contrast of ~7.2:1, still demonstrating the great BDTE performance. Moreover, the experimental non-polarized average emissivity displays $\bar{e}_{max}\sim$0.4070 (at 74°) and $\bar{e}_{min}\sim$0.0548 (at 16°) (corresponding to an emissivity contrast of ~7.4:1), exhibiting the great potential in BDTE applications.


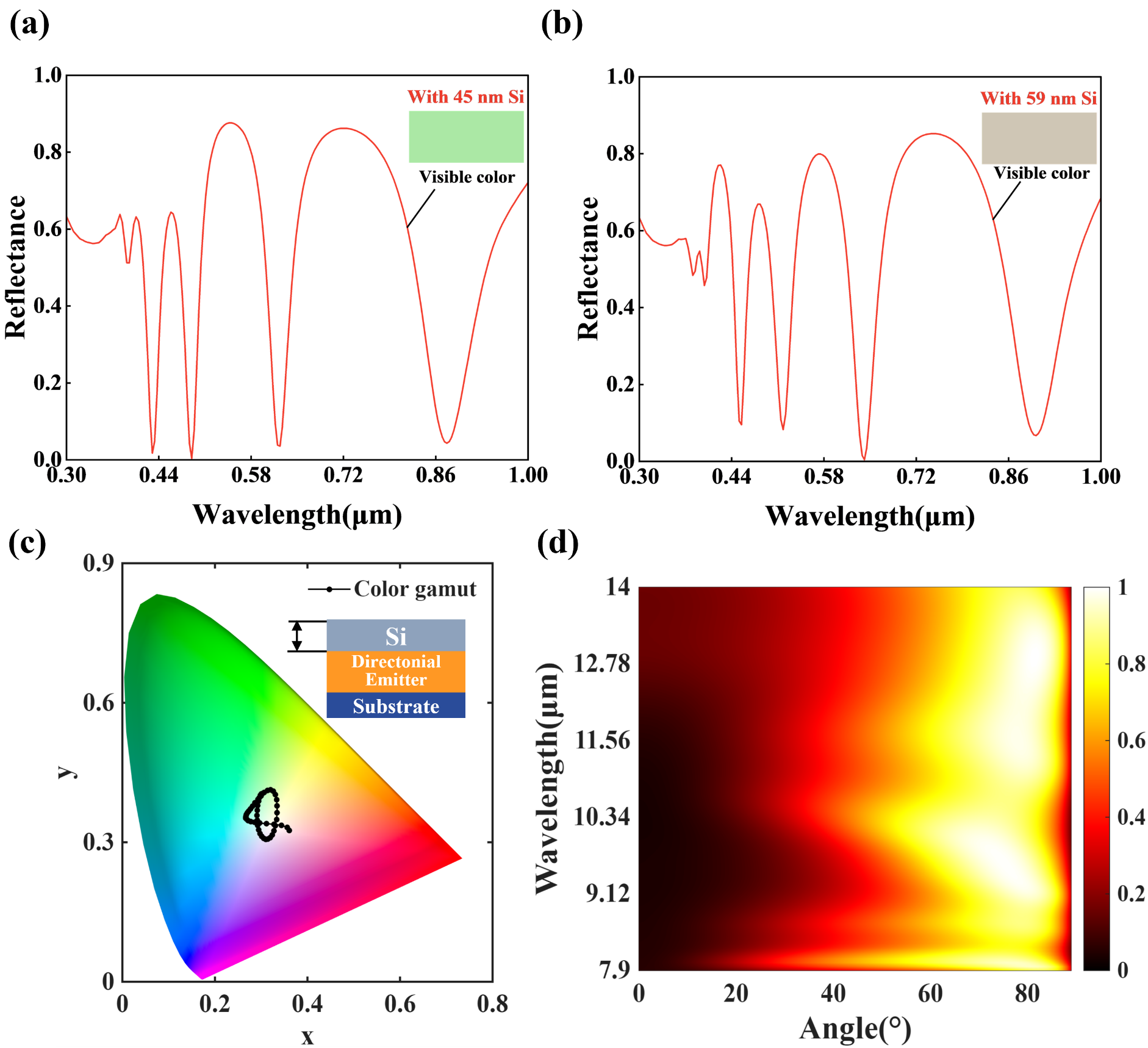


**Figure S18.** Reflectivity spectra and visible colors of the emitter with a) 45 nm-thick and b) 59 nm-thick Si layers on top within the wavelength range of 0.3-1 μm. c) The color gamut of the directional emitter with Si layers on top (top Si layer: 1-100 nm). d) The calculated angle-resolved emissivity spectrum of the optimal emitter with a 100 nm-thick Si layer on top under p-polarization.

As shown in **Figure S18**a and **S18**b, our structure enables the color to be tuned to green and brown, which can be utilized to camouflage as grasslands and tree branches, respectively, demonstrating great potential for visible camouflage. Moreover, varying the thickness of the top Si layer (1–100 nm) enables tuning the structure’s color within a specific gamut (**Figure S18**c), which facilitates applications in more practical scenarios. Notably, although the addition of a Si layer reduces the directional emission performance to some extent, the 100 nm-thick Si layer stacked on top of our emitter still exhibits favorable BDTE (FOM~7.2) in the 7.9-14 μm range (**Figure S18**d). Therefore, the structure can simultaneously realize color tuning and retain high BDTE performance for a top Si layer with a thickness up to 100 nm.

**Reference**

[1] L. A. A. Pettersson, L. S. Roman, O. Inganäs, *Journal of Applied Physics* **1999**, *86*, 487.

[2] M. Chikhi, F. Benkabou, *Plasmonics* **2015**, *10*, 1467.

[3] K. Witt, in *Colorimetry*, **2007**, pp. 79–100.

[4] W. S. Mokrzycki, M. Tatol, *MG&V* **2011**, *20*, 383.

[5] A. M. Hofmeister, E. Keppel, A. K. Speck, *Monthly Notices of the Royal* *Astronomical Society* **2003**, *345*, 16.

[6] T. J. Bright, J. I. Watjen, Z. M. Zhang, C. Muratore, A. A. Voevodin, D. I. Koukis, D. B. Tanner, D. J. Arenas, *Journal of Applied Physics* **2013**, *114*, 083515.

[7] J. Kischkat, S. Peters, B. Gruska, M. Semtsiv, M. Chashnikova, M. Klinkmüller, O. Fedosenko, S. Machulik, A. Aleksandrova, G. Monastyrskyi, Y. Flores, W. Ted Masselink, *Appl. Opt.* **2012**, *51*, 6789.
